# Glycerolipidome responses to freezing- and chilling-induced injuries: examples in *Arabidopsis* and rice

**DOI:** 10.1186/s12870-016-0758-8

**Published:** 2016-03-22

**Authors:** Guowei Zheng, Lixia Li, Weiqi Li

**Affiliations:** Key Laboratory for Plant Diversity and Biogeography of East Asia, Kunming Institute of Botany, Chinese Academy of Sciences, Kunming, Yunnan 650202 People’s Republic of China; Germplasm Bank of Wild Species, Kunming Institute of Botany, Chinese Academy of Sciences, Kunming, 650201 People’s Republic of China; Guiyang Medicinal Botanical Garden, Guiyang, 550002 People’s Republic of China

**Keywords:** Chilling, Freezing, *Arabidopsis*, Rice, Glycerolipidome

## Abstract

**Background:**

Glycerolipids are the principal constituent of cellular membranes; remodelling of glycerolipids plays important roles in temperature adaptation in plants. Temperate plants can endure freezing stress, but even chilling at above-zero temperatures can induce death in tropical species. However, little is known about the differences in glycerolipid response to low temperatures between chilling-sensitive and freezing-tolerant plants. Using ESI-MS/MS-based lipidomic analysis, we compared the glycerolipidome of chilling (4 and 10 °C)-treated rice with that of freezing (−6 and −12 °C)-treated *Arabidopsis*, both immediately after these low-temperature treatments and after a subsequent recovery culture period.

**Results:**

*Arabidopsis* is a 16:3 plant that harbours both eukaryotic and prokaryotic-type lipid synthesis pathways, while rice is an 18:3 plant that harbours only the eukaryotic lipid synthesis pathway. *Arabidopsis* contains higher levels of galactolipids than rice and has a higher double bond index (DBI). *Arabidopsis* contains lower levels of high melting point phosphatidylglycerol (PG) molecules and has a lower average acyl chain length (ACL). Marked phospholipid degradation occurred during the recovery culture period of non-lethal chilling treated rice, but did not occur in non-lethal freezing treated *Arabidopsis*. Glycerolipids with larger head groups were synthesized more in *Arabidopsis* than in rice at sub-lethal low-temperatures. Levels of phosphatidic acid (PA) and phosphatidylinositol (PI) rose in both plants after low-temperature treatment. The DBI and ACL of total lipids did not change during low-temperature treatment.

**Conclusions:**

A higher DBI and a lower ACL could make the membranes of *Arabidopsis* more fluid at low temperatures. The ability to synthesize glycerolipids containing a larger head group may correlate with low-temperature tolerance. The low-temperature-induced increase of PA may play a dual role in plant responses to low temperatures: as a lipid signal that initiates tolerance responses, and as a structural molecule that, on extensive in large accumulation, could damage the integrity of membranes. Changes in ACL and DBI are responses of plants to long-term low temperature.

**Electronic supplementary material:**

The online version of this article (doi:10.1186/s12870-016-0758-8) contains supplementary material, which is available to authorized users.

## Background

Low-temperature injury is one of the most important factors causing substantial agricultural losses. The ability of plants to tolerate low temperatures is an important determinant of their productivity and geographical distribution. The climate of the area from which a plant originates has a major influence on its sensitivity to freezing and chilling stresses [1]. Many temperate plants can tolerate sub-zero temperatures (freezing), and, after exposure to low temperatures (usually 4 °C) for a period, they develop increased freezing tolerance [2]. However, in some plants from tropical or subtropical areas, such as rice, exposure even to low temperatures above 0 °C (chilling) can induce severe damage [3]. Freezing-induced injury to plants differs from that associated with chilling, because of the presence of ice inside plant tissues. As a result, there are major differences in the physiological, biochemical and molecular responses of plants to freezing and chilling stresses [4–7]. For example, chilling tolerance in pea (*Pisum sativum*) was suggested to be related to an increase in proteins that are involved in osmotic adjustment and antioxidative responses; however, freezing tolerance seemed to depend on proteins that maintain the stability of the photosystems as well as the capacity for photosynthesis [5].

Membranes are integral to the structure and function of all cells and are very sensitive to environmental changes. The responses of membranes to temperature changes have been investigated extensively and results indicate that membrane damage is the major cause of low-temperature-induced injury in plants [8–10]. Phospholipids and galactolipids are the main glycerolipids, and are respectively the major constituents of plasma and chloroplast membranes. Regulation of the level of saturation and the constitution of glycerolipids is important for plants to maintain the integrity and fluidity of their membranes under temperature stresses [11–13]. After introduction into a warmer lowland area from an alpine region, the level of saturation in all measured phospholipids of *Meconopsis racemosa* leaves increased [14]. Changing the level of saturation of glycerolipids has been identified as a strategy used by plants to cope with both long-term high and low temperatures; in contrast, upon short-term temperature stresses, the level of saturation of glycerolipids was maintained [15]. In addition to reducing the level of saturation of glycerolipids, adjustment of the composition of lipids to balance the ratio of bilayer- to non-bilayer-forming membrane is also important for plant responses to low temperatures. At low temperatures, the proportion of phospholipids tends to increase [13, 16]. Glycerolipids harbouring large polar head groups, such as digalactosyldiacylglycerol (DGDG) and phosphatidylcholine (PC), are bilayer lipids that may increase membrane stability. In contrast, monogalactosyldiacylglycerol (MGDG) and phosphatidylethanolamine (PE), which have relatively small head groups, show a higher propensity for transition to non-bilayer H_II_-type structures [17, 18]. Increases in the ratio of bilayer- to non-bilayer-forming membrane lipids, for example PC:PE and DGDG:MGDG, have been observed in plants responding to low temperatures [18, 19]. Leaves of *Brassica napus* showed a tendency to synthesise more desaturated PC and MGDG at low temperatures [20]. Plants that have different kinds of MGDG molecules have different pathways for synthesizing lipids: plants that have only 36:6 (total acyl carbons:total double bonds) MGDG molecules are 18:3 plants, which only have the eukaryotic-type lipid synthesis pathway; plants that contain both 34:6 and 36:6 MGDG molecules are 16:3 plants, which have both prokaryotic and eukaryotic-type lipid synthesis pathways [21]. However, little is known about the relationships between the different pathways for synthesizing lipids in plants that are tolerant to low temperatures.

Most studies on changes in the lipidome in response to low temperatures used the model plant *Arabidopsis*, and investigated its freezing tolerance [12, 13, 18, 22]. However, freezing-induced membrane damage may differ from that associated with chilling, because cells can suffer different types of injury. The lipidome responses of plants upon chilling-induced injury or even death have scarcely been studied; to the best of our knowledge, only Li et al. have focused on this issue, studying the *Jatropha curcas* lipidome response to chilling treatment [23].

Rice is a chilling-sensitive plant that dies after the temperature falls to 4 °C [3]. In contrast, after acclimation to cold at 4 °C, *Arabidopsis* can tolerate freezing temperatures as low as −6 °C [24]. We are interested in the responses of the lipidomes of plants that have different levels of tolerance to low temperatures in terms of chilling- and freezing-induced injury. Here, we report an investigation of the responses of the glycerolipidomes of rice and *Arabidopsis* after exposure of the plants to chilling (4 and 10 °C) and freezing (−6 and −12 °C) temperatures, respectively. Our principal aims were to address two main questions: (i) do different lipidome profiles contribute to the different capacities of *Arabidopsis* and rice to tolerate low temperatures? (ii) what are the differences in lipidome response between *Arabidopsis* and rice during treatment at different low temperatures?

## Results

We used an ESI-MS/MS-based lipid profiling approach [25] to investigate changes in *Arabidopsis* and rice leaf lipid profiles during low-temperature treatments at above and below 0 °C. This lipidomic approach allowed us to profile 11 lipid classes, which contained 150 molecular species of membrane glycerolipids, including two classes of galactolipids, six of phospholipids and three of lysophospholipids (lysoPLs). For sub-zero low-temperature (freezing) treatment, cold-acclimated (4 °C for 3 days) *Arabidopsis* was treated at non-lethal (−6 °C) and lethal (−12 °C) sub-zero temperatures. For low-temperature treatment above 0 °C (chilling), rice seedlings were treated at 10 or 4 °C for 6 days, which represented non-lethal and lethal treatments, respectively. For *Arabidopsis* and rice respectively, plants treated at −6 and 10 °C would survive after 3 days of recovery from low-temperature treatment, whereas plants treated at −12 and 4 °C would die after this period. Leaf lipid profiles were analysed immediately after low-temperature treatment and after subsequent recovery culture for 1 and 3 days. Five independent biological replicates were analysed for each treatment.

### Different lipid profiles between cold-acclimated *Arabidopsis* and rice

In the first analysis, we compared the lipid profile of cold-acclimated *Arabidopsis* with that of rice grown in normal conditions (22/18 °C, day/night). Galactolipids and phospholipids are the main components of the glycerolipids in plants. Among the glycerolipids that we tested, the level of galactolipids was higher in *Arabidopsis* than in rice; the ratio of galactolipids to phospholipids was 8.83 and 6.46 in *Arabidopsis* and rice respectively (Table 1). MGDG and DGDG are the main extraplastidic lipids, while PC and PE are the main plastidic lipids. These lipid classes have differently sized head groups and play different roles in the integrity of membranes under stresses. The ratio of MGDG/DGDG in cold-acclimated *Arabidopsis* was 4.97, whereas in normal growth rice it was 2.67. The ratio of PC/PE in *Arabidopsis* was 6.47, whereas in rice it was 14.09 (Table 1).

**Table 1:** Total amount of lipid in each head-group class and ratios of different lipid classes after various low-temperature treatments of *Arabidopsis* and rice

Lipid class	Plant species		*Arabidopsis* (−6 °C)/rice (10 °C)			*Arabidopsis* (−12 °C)/rice (4 °C)		
		C	T	R1	R3	T	R1	R3
		Lipid content (nmol per mg dry weight)						
DGDG	*Arabidopsis*	47.49 ± 3.00^ab^	50.54 ± 4.68^a^	41.28 ± 3.71^c^	45.22 ± 7.03^bc^	17.17 ± 2.12^d^	5.88 ± 1.01^e^	3.41 ± 1.25^e^
	Rice	38.69 ± 2.29^ab^	39.07 ± 3.08^ab^	38.22 ± 2.19^b^	41.58 ± 1.12^a^	27.91 ± 3.46^c^	22.61 ± 1.70^d^	8.43 ± 1.12^e^
MGDG	*Arabidopsis*	235.54 ± 17.77^a^	159.59 ± 26.35^b^	174.79 ± 24.64^b^	213.70 ± 24.43^a^	7.68 ± 1.02^c^	3.93 ± 0.65^c^	2.82 ± 0.62^c^
	Rice	103.43 ± 5.93^a^	73.53 ± 4.96^c^	70.97 ± 3.83^c^	93.72 ± 4.07^b^	51.03 ± 11.24^d^	22.28 ± 2.44^e^	6.47 ± 2.54^f^
PG	*Arabidopsis*	11.12 ± 3.13^ab^	11.77 ± 1.87^a^	9.56 ± 1.01^b^	9.68 ± 1.32^b^	4.97 ± 0.75^c^	2.36 ± 0.42^d^	1.42 ± 0.17^d^
	Rice	5.46 ± 0.88^a^	3.05 ± 0.28^b^	2.72 ± 0.25^bc^	2.99 ± 0.38^bc^	2.47 ± 0.43^c^	1.45 ± 0.16^d^	0.54 ± 0.09^e^
PA	*Arabidopsis*	0.25 ± 0.07^d^	2.32 ± 0.83^cd^	1.41 ± 0.69^d^	0.50 ± 0.10^d^	24.75 ± 4.13^a^	20.74 ± 5.88^b^	6.00 ± 3.24^c^
	Rice	0.18 ± 0.07^c^	0.47 ± 0.09^c^	0.11 ± 0.02^c^	0.12 ± 0.03^c^	3.57 ± 1.23^b^	5.90 ± 1.26^a^	2.81 ± 0.27^b^
PC	*Arabidopsis*	13.80 ± 1.34^b^	13.52 ± 2.35^b^	16.51 ± 1.79^a^	15.94 ± 1.07^a^	3.18 ± 0.86^c^	0.68 ± 0.52^d^	0.76 ± 0.43^d^
	Rice	12.66 ± 0.94^a^	10.63 ± 0.72^b^	9.77 ± 0.37^bc^	9.13 ± 0.73^c^	9.84 ± 1.22^bc^	6.30 ± 0.37^d^	2.57 ± 0.59^e^
PE	*Arabidopsis*	2.18 ± 0.39^a^	2.15 ± 0.81^a^	2.66 ± 0.99^a^	2.50 ± 0.76^a^	0.36 ± 0.16^b^	0.09 ± 0.07^b^	0.07 ± 0.05^b^
	Rice	0.97 ± 0.21^b^	1.41 ± 0.17^a^	1.03 ± 0.15^b^	0.76 ± 0.12^c^	1.11 ± 0.15^b^	0.70 ± 0.05^c^	0.25 ± 0.08^d^
PI	*Arabidopsis*	5.21 ± 0.65^b^	6.93 ± 1.59^a^	6.90 ± 1.35^a^	6.92 ± 0.56^a^	5.68 ± 0.59^b^	3.31 ± 0.31^c^	1.06 ± 0.24^d^
	Rice	2.61 ± 0.22^ab^	2.46 ± 0.19^b^	2.47 ± 0.24^b^	2.48 ± 0.26^b^	1.72 ± 0.05^c^	2.84 ± 0.23^a^	1.56 ± 0.32^c^
PS	*Arabidopsis*	0.21 ± 0.06^ab^	0.09 ± 0.03^c^	0.18 ± 0.04^b^	0.25 ± 0.04^a^	0.03 ± 0.01^d^	0.02 ± 0.01^d^	0.03 ± 0.03^d^
	Rice	0.22 ± 0.03^a^	0.15 ± 0.03^bc^	0.11 ± 0.01^de^	0.13 ± 0.02^cd^	0.17 ± 0.03^b^	0.14 ± 0.01^cd^	0.07 ± 0.03^e^
Total	*Arabidopsis*	315.92 ± 16.69^a^	247.16 ± 27.43^b^	253.44 ± 29.78^b^	294.83 ± 33.53^a^	64.20 ± 5.07^c^	37.37 ± 6.16^cd^	15.83 ± 3.84^d^
	Rice	164.27 ± 7.38^a^	130.88 ± 8.70^c^	125.46 ± 6.23^c^	149.96 ± 5.93^b^	98.00 ± 15.14^d^	62.43 ± 3.88^e^	22.82 ± 3.74^f^
		Ratio of lipid						
MGDG:DGDG	*Arabidopsis*	4.97 ± 0.48^a^*	3.20 ± 0.71^c^*	4.23 ± 0.46^b^*	4.75 ± 0.31^ab^*	0.46 ± 0.09^d^*	0.67 ± 0.07^d^*	0.91 ± 0.37^d^
	Rice	2.67 ± 0.10^a^	1.88 ± 0.08^c^	1.86 ± 0.06^c^	2.23 ± 0.08^b^	1.81 ± 0.20^c^	0.99 ± 0.10^d^	0.75 ± 0.23^e^
PC:PE	*Arabidopsis*	6.47 ± 1.09^c^*	6.93 ± 2.33^c^	5.46 ± 0.94^c^*	6.89 ± 2.10^c^*	8.31 ± 0.91^b^	9.30 ± 4.93^a^	13.48 ± 4.07^a^
	Rice	14.09 ± 0.57^a^	7.57 ± 0.74^e^	9.53 ± 1.02^cd^	12.20 ± 1.46^b^	8.92 ± 0.29^de^	9.10 ± 0.88^d^	10.67 ± 1.03^c^
Galactolipid:Phospholipid	*Arabidopsis*	8.83 ± 1.64^a^*	5.79 ± 0.87^c^	5.81 ± 0.55^c^*	7.23 ± 0.52^b^*	0.65 ± 0.10^d^*	0.38 ± 0.12^d^*	0.78 ± 0.39^d^*
	Rice	6.46 ± 0.60^b^	6.19 ± 0.24^b^	6.75 ± 0.38^b^	8.63 ± 0.44^a^	4.17 ± 0.67^c^	2.61 ± 0.33^d^	1.96 ± 0.61^e^

“C” represents “control”, “T” indicates samples taken immediately after each cold treatment, and “R1” and “R3” indicate samples taken after post treatment recovery culture for 1 and 3 days, respectively. Values in the same row marked with different letters are significantly different, while an asterisk means that the value in Arabidopsis is significantly different from that in rice (*p* < 0.05). Values are means ± S.D. (*n* = 4 or 5)

**Fig. 1:**
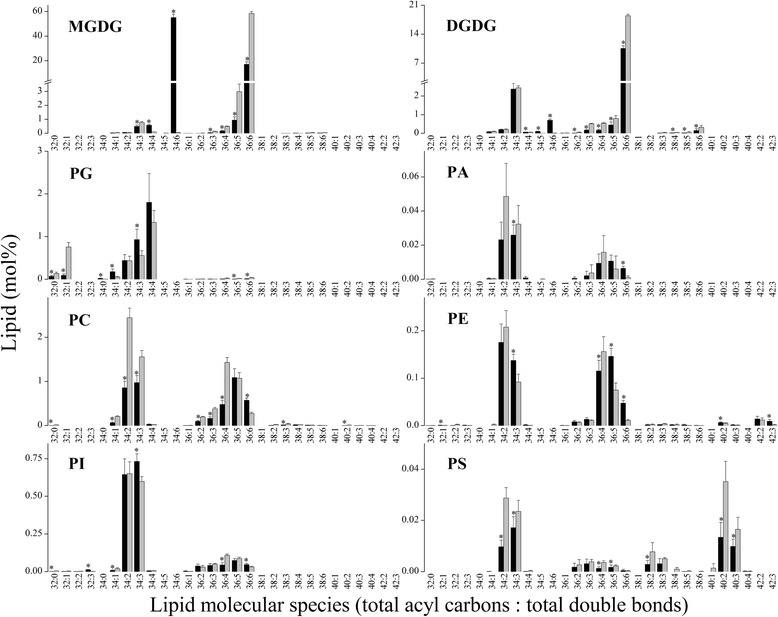
Compositions of lipid molecular species in cold acclimated *Arabidopsis* (*black bars*) and normal growth rice (*light grey bars*). The bars show relative content (mol%) values for all measured samples. An asterisk shows that the value in *Arabidopsis* is significantly different from that in rice (*p* < 0.05). Values are means ± S.D. (*n* = 5)

From detailed comparative analysis of the lipids between the two plants, we found that *Arabidopsis* contained both 34:6 and 36:6 MGDG. However, rice contained only 36:6 MGDG molecules (Fig. 1; Additional file 1: Table S1). This difference shows that the two plants have different lipid synthesis pathways; *Arabidopsis* is a 16:3 plant that has both eukaryotic and prokaryotic-type lipid synthesis pathways, while rice is an 18:3 plant that has only the eukaryotic-type lipid synthesis pathway [21]. Some of the PG molecules with compositions of 16:0/16:0 and 16:0/16:1, such as 32:0 and 32:1 PG, respectively, have high melting points. The levels of these PG molecules correlate positively with the cold sensitivity of plants [26, 27]. The content of 32:0 PG in rice was found to be almost double that in *Arabidopsis*. In addition, the content of 32:1 PG was eight times greater in rice than in *Arabidopsis* (Fig. 1).

The ACL and DBI of glycerolipids are two determinants of membrane fluidity. To understand membrane fluidity under low-temperature stresses, we compared the ACL and DBI between *Arabidopsis* and rice. Fatty acids with more than 18 carbon atoms per chain are known as very long chain fatty acids (VLCFAs) [28]. Glycerolipids that have acyl chains with more than 36 carbons, such as MGDG, DGDG, PC, PE and PS, contain VLCFAs (Fig. 1). The relative content of galactolipids with an acyl chain that has > 36 carbons was found to be 0.198 % and 0.401 % in *Arabidopsis* and rice, respectively, while for phospholipids the corresponding values were 0.125 % and 0.205 %, respectively. By calculating the acyl chain length of eight kinds of glycerolipid, we found that the ACL of total lipids was 34.66 in cold-acclimated *Arabidopsis* and 35.73 in normal growth rice (Table 2). The fact that the ACL of galactolipids is lower in *Arabidopsis* than in rice may contribute to the lower ACL of total lipids in *Arabidopsis*, because galactolipids constitute > 80 % of the total lipids. In addition, the ACL of MGDG in *Arabidopsis* was 1.48 carbons less than that in rice. However, the ACL of most phospholipids, except for PS, in *Arabidopsis* was greater than that in rice (Table 2). The DBI is the average number of double bonds in the fatty acid chains of a glycerolipid molecular species; a high DBI indicates the presence of more highly unsaturated membrane lipids, and vice versa. For all glycerolipid classes, the DBI of *Arabidopsis* differs from that of rice (except for PI, which did not show any difference between the two plants). The levels of the DBI of most lipid classes, including total lipids, except for DGDG, were higher in *Arabidopsis* than in rice (Table 3).

**Table 2:** Acyl chain length (ACL) of membrane lipids in *Arabidopsis* and rice after various low-temperature treatments

Lipid class	Plant species		*Arabidopsis* (−6 °C)/Rice (10 °C)			*Arabidopsis* (−12 °C)/Rice (4 °C)		
		C	T	R1	R3	T	R1	R3
		Acyl chain length (ACL)						
DGDG	*Arabidopsis*	35.56 ± 0.02^bc^*	35.59 ± 0.08^abc^*	35.61 ± 0.04^ab^*	35.63 ± 0.03^a^*	35.54 ± 0.03^c^*	35.47 ± 0.02^d^*	35.36 ± 0.06^e^*
	Rice	35.80 ± 0.02^b^	35.84 ± 0.03^a^	35.80 ± 0.03^b^	35.73 ± 0.02^c^	35.78 ± 0.02^b^	35.87 ± 0.04^a^	35.85 ± 0.04^a^
MGDG	*Arabidopsis*	34.49 ± 0.06^e^*	34.54 ± 0.08^de^*	34.60 ± 0.05^cd^*	34.57 ± 0.03^de^*	34.65 ± 0.06^bc^*	34.80 ± 0.08^a^*	34.73 ± 0.07^ab^*
	Rice	35.97 ± 0.00^ab^	35.98 ± 0.00^a^	35.97 ± 0.00^b^	35.95 ± 0.00^c^	35.98 ± 0.00^a^	35.98 ± 0.01^a^	35.95 ± 0.01^c^
PG	*Arabidopsis*	33.92 ± 0.02^ab^*	33.93 ± 0.01^a^*	33.91 ± 0.02^ab^*	33.88 ± 0.01^c^*	33.92 ± 0.01^ab^*	33.90 ± 0.01^b^*	33.85 ± 0.03^d^*
	Rice	33.51 ± 0.05^a^	33.46 ± 0.01^ab^	33.42 ± 0.03^bc^	33.41 ± 0.02^bc^	33.52 ± 0.02^a^	33.39 ± 0.05^c^	33.20 ± 0.13^d^
PA	*Arabidopsis*	34.73 ± 0.09^d^*	35.00 ± 0.11^a^	34.87 ± 0.02^c^	34.84 ± 0.05^c^	34.98 ± 0.02^ab^*	34.90 ± 0.05^bc^*	34.73 ± 0.08^d^
	Rice	34.44 ± 0.19^c^	34.93 ± 0.05^a^	34.78 ± 0.17^b^	34.74 ± 0.14^b^	34.73 ± 0.02^b^	34.76 ± 0.03^b^	34.67 ± 0.04^b^
PC	*Arabidopsis*	35.15 ± 0.07^a^*	35.21 ± 0.07^a^*	35.09 ± 0.04^abc^*	34.94 ± 0.02^c^*	35.13 ± 0.10^ab^	34.98 ± 0.12^bc^	35.10 ± 0.27^abc^
	Rice	34.93 ± 0.03^c^	34.95 ± 0.01^c^	34.81 ± 0.01^d^	34.85 ± 0.04^d^	35.08 ± 0.04^a^	35.00 ± 0.02^b^	35.07 ± 0.07^a^
PE	*Arabidopsis*	35.36 ± 0.09^abc^*	35.74 ± 0.23^a^*	35.33 ± 0.12^bc^*	35.21 ± 0.12^c^	35.67 ± 0.26^ab^*	35.17 ± 0.58^c^	35.11 ± 0.33^c^
	Rice	35.15 ± 0.05^a^	35.06 ± 0.06^b^	35.07 ± 0.05^ab^	35.12 ± 0.01^ab^	35.14 ± 0.07^a^	35.06 ± 0.08^b^	34.96 ± 0.09^c^
PI	*Arabidopsis*	34.28 ± 0.04^ab^*	34.32 ± 0.03^a^*	34.17 ± 0.03^cd^*	34.13 ± 0.02^d^*	34.31 ± 0.03^ab^*	34.26 ± 0.05^b^*	34.20 ± 0.06^c^*
	Rice	34.37 ± 0.01^d^	34.50 ± 0.03^b^	34.27 ± 0.03^e^	34.24 ± 0.02^e^	34.67 ± 0.03^a^	34.41 ± 0.06^c^	34.35 ± 0.02^d^
PS	*Arabidopsis*	36.92 ± 0.30^ab^	37.07 ± 0.56^a^	35.95 ± 0.47^bc^*	36.63 ± 0.38^abc^*	36.24 ± 0.63^abc^	35.77 ± 0.76^c^*	36.87 ± 1.63^ab^
	Rice	37.11 ± 0.24^bc^	37.32 ± 0.30^ab^	37.57 ± 0.29^a^	37.35 ± 0.17^ab^	36.44 ± 0.30^e^	36.84 ± 0.26^cd^	36.51 ± 0.38^de^
Total	*Arabidopsis*	34.66 ± 0.05^b^*	34.76 ± 0.07^ab^*	34.76 ± 0.03^ab^*	34.72 ± 0.01^b^*	34.86 ± 0.02^a^*	34.70 ± 0.10^b^*	34.46 ± 0.15^c^*
	Rice	35.73 ± 0.02^a^	35.74 ± 0.01^a^	35.72 ± 0.01^a^	35.73 ± 0.01^a^	35.65 ± 0.03^b^	35.52 ± 0.03^c^	35.36 ± 0.09^d^

ACL = (∑[*n* × mol % lipid])/100, *n* is the total number of carbons in the two fatty acid chains of each glycerolipid molecule. “C” represents “control”, “T” indicates samples taken immediately after each cold treatment, and “R1” and “R3” indicate samples taken after post treatment recovery culture for 1 and 3 days, respectively. Values in the same row marked with different letters are significantly different, while an asterisk means that the value in *Arabidopsis* is significantly different from that in rice (*p* < 0.05). Values are means ± SD (*n* = 4 or 5)

**Table 3:** Double-bond index (DBI) of membrane lipids in *Arabidopsis* and rice after various low-temperature treatments

Lipid class	Plant species		*Arabidopsis* (−6 °C)/Rice (10 °C)			*Arabidopsis* (−12 °C)/Rice (4 °C)		
		C	T	R1	R3	T	R1	R3
		Double-bond index (DBI)						
DGDG	*Arabidopsis*	5.34 ± 0.04^b^*	5.41 ± 0.13^ab^*	5.47 ± 0.05^a^	5.49 ± 0.03^a^*	5.41 ± 0.04^ab^*	5.33 ± 0.06^b^*	5.08 ± 0.14^c^*
	Rice	5.47 ± 0.02^b^	5.56 ± 0.02^a^	5.46 ± 0.01^b^	5.34 ± 0.02^c^	5.49 ± 0.02^b^	5.59 ± 0.03^a^	5.49 ± 0.06^b^
MGDG	*Arabidopsis*	5.94 ± 0.01^a^*	5.93 ± 0.02^a^*	5.89 ± 0.01^b^*	5.92 ± 0.03^ab^*	5.93 ± 0.02^ab^	5.81 ± 0.04^c^*	5.74 ± 0.05^d^*
	Rice	5.89 ± 0.01^b^	5.89 ± 0.01^b^	5.83 ± 0.01^cd^	5.84 ± 0.01^c^	5.92 ± 0.00^a^	5.90 ± 0.01^b^	5.82 ± 0.03^d^
PG	*Arabidopsis*	3.16 ± 0.08^a^*	3.15 ± 0.11^a^*	3.09 ± 0.08^a^*	3.09 ± 0.10^a^*	3.11 ± 0.04^a^*	3.12 ± 0.06^a^*	2.97 ± 0.08^b^*
	Rice	2.71 ± 0.04^a^	2.56 ± 0.02^b^	2.40 ± 0.05^c^	2.39 ± 0.04^c^	2.58 ± 0.08^b^	2.35 ± 0.11^c^	2.20 ± 0.10^d^
PA	*Arabidopsis*	3.36 ± 0.13^d^*	4.24 ± 0.23^a^*	3.81 ± 0.12^b^*	3.58 ± 0.16^c^*	3.82 ± 0.07^b^*	3.77 ± 0.04^b^*	3.39 ± 0.08^d^*
	Rice	2.79 ± 0.18^c^	3.16 ± 0.10^b^	2.93 ± 0.20^c^	2.95 ± 0.19^c^	3.32 ± 0.06^ab^	3.42 ± 0.05^a^	3.21 ± 0.06^b^
PC	*Arabidopsis*	3.77 ± 0.04^ab^*	4.03 ± 0.15^a^*	3.84 ± 0.11^ab^*	3.73 ± 0.06^ab^*	3.73 ± 0.13^ab^*	3.54 ± 0.09^b^*	3.60 ± 0.58^b^
	Rice	3.17 ± 0.02^c^	3.17 ± 0.01^c^	3.03 ± 0.04^d^	3.05 ± 0.04^d^	3.36 ± 0.03^a^	3.28 ± 0.06^b^	3.26 ± 0.06^b^
PE	*Arabidopsis*	3.51 ± 0.08^ab^*	3.65 ± 0.11^a^*	3.51 ± 0.12^ab^*	3.49 ± 0.05^abc^*	3.29 ± 0.09^c^*	2.99 ± 0.18^d^	3.39 ± 0.29^bc^*
	Rice	3.17 ± 0.03^b^	3.12 ± 0.02^b^	3.18 ± 0.03^b^	3.26 ± 0.05^a^	3.12 ± 0.02^b^	3.00 ± 0.08^c^	3.03 ± 0.07^c^
PI	*Arabidopsis*	2.78 ± 0.02^b^	2.99 ± 0.08^a^*	2.82 ± 0.07^b^*	2.79 ± 0.04^b^*	2.78 ± 0.03^b^*	2.80 ± 0.01^b^*	2.65 ± 0.10^c^*
	Rice	2.78 ± 0.04^c^	2.86 ± 0.03^b^	2.61 ± 0.03^d^	2.60 ± 0.01^d^	3.19 ± 0.06^a^	2.90 ± 0.06^b^	2.79 ± 0.03^c^
PS	*Arabidopsis*	2.62 ± 0.06^a^*	2.74 ± 0.12^a^*	2.74 ± 0.08^a^*	2.64 ± 0.08^a^*	2.78 ± 0.26^a^*	2.71 ± 0.09^a^*	2.49 ± 0.59^a^
	Rice	2.47 ± 0.03^a^	2.41 ± 0.10^ab^	2.41 ± 0.05^ab^	2.45 ± 0.05^a^	2.47 ± 0.10^a^	2.34 ± 0.08^b^	2.43 ± 0.05^ab^
Total	*Arabidopsis*	5.58 ± 0.04^a^*	5.46 ± 0.06^b^*	5.46 ± 0.02^b^*	5.54 ± 0.05^ab^*	4.33 ± 0.07^c^*	4.08 ± 0.11^d^*	4.08 ± 0.15^d^*
	Rice	5.40 ± 0.04^a^	5.39 ± 0.02^a^	5.33 ± 0.02^ab^	5.39 ± 0.01^a^	5.26 ± 0.07^b^	5.01 ± 0.07^c^	4.71 ± 0.17^d^

DBI = (∑[*N* × mol % lipid])/100, *N* is the total number of double bonds in the two fatty acid chains of each glycerolipid molecule. “C” represents “control”, “T” indicates samples taken immediately after each cold treatment, and “R1” and “R3” indicate samples taken after post treatment recovery for 1 and 3 days, respectively. Values in the same row marked with different letters are significantly different, while an asterisk means that the value in *Arabidopsis* is significantly different from that in rice (*p* < 0.05). Values are means ± SD (*n* = 4 or 5)

### Changes in membrane lipid content distinguished lethal from sub-lethal low-temperature treated plants

**Fig. 2:**
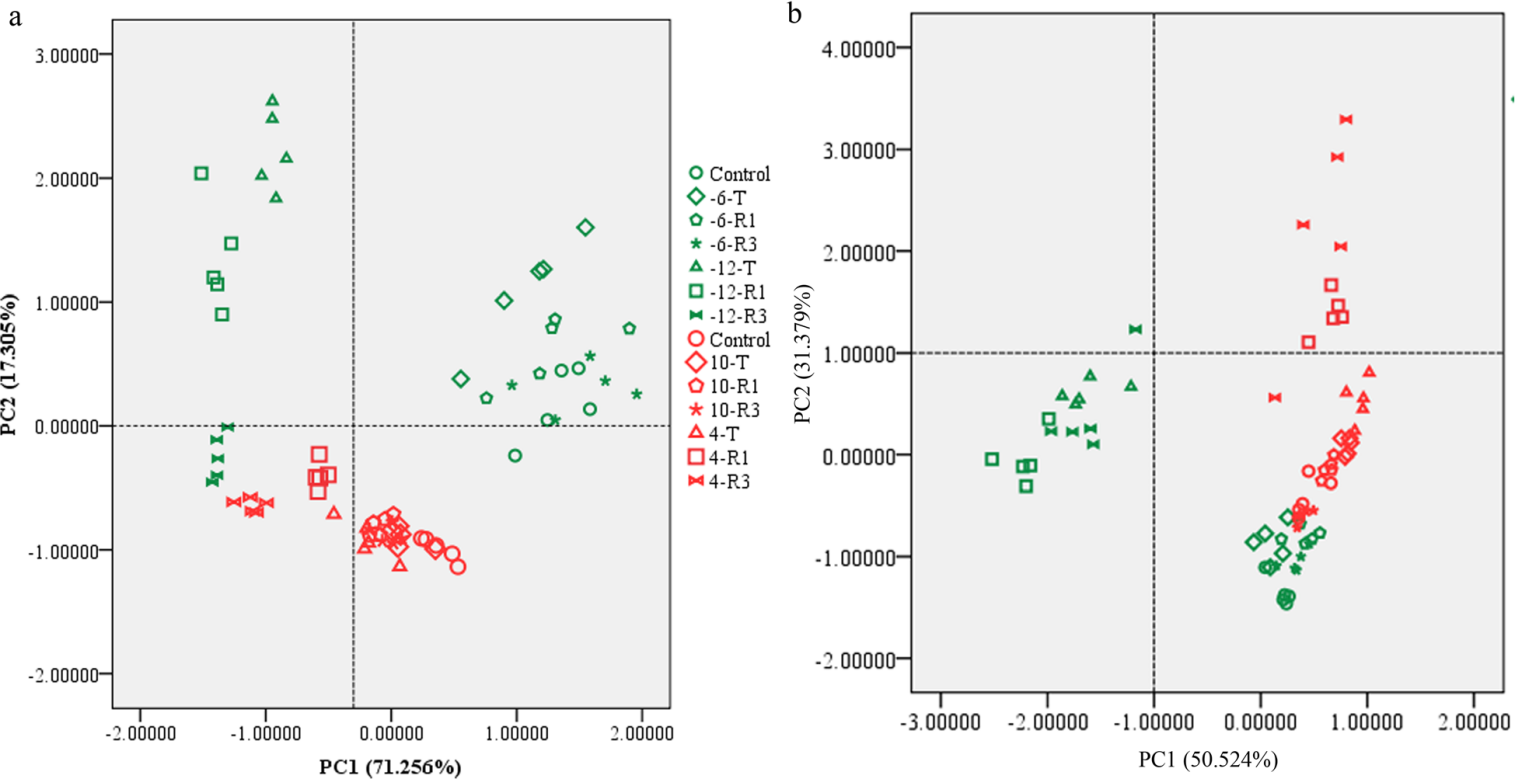
Principal component analysis (PCA) of the lipid composition upon different low-temperature treatments of *Arabidopsis* and rice. **a** PCA analysis of lipid content (nmol per mg). **b** PCA analysis of lipid relative content (mol%). Green and red symbols represent *Arabidopsis* and rice, respectively. The different treatments are represented by different shapes. −6, −12, 10 and 4 represent temperatures of treatment in °C. −6 °C and 10 °C are non-lethal low temperatures for *Arabidopsis* and rice, while −12 °C and 4 °C are lethal low temperatures for *Arabidopsis* and rice, respectively. T represents samples immediately after low-temperature treatment, R1 and R3 represent samples after 1 and 3 days of recovery growth

The effects of different low-temperature treatments on the glycerolipidome and their relationships were clarified using principal component analysis (PCA). In the PCA analysis of absolute levels of glycerolipids, the two principal components explained 71 % and 17 % of the overall variance (Fig. 2a). The loadings of PC1 identified MGDG, DGDG, PC and PE as being most important for the separation, whereas for PC2, phosphatidic acid (PA) and PI were identified (Additional file 1: Table S2). The PCA separated *Arabidopsis* and rice efficiently; it also separated lethal and sub-lethal low-temperature treated plants (Fig. 2a). Loadings of PC1 represented the most abundant glycerolipids in plants; the separation of different samples along this axis may indicate low-temperature induced lipid degradation. PC2 represented the two signalling lipids, PA and PI. Along this axis, *Arabidopsis* and rice were separated, which may indicate that different lipid signals occur during low-temperature treatment of these two plants. To compare the compositional variability and inter-conversion of membrane lipids, we performed a PCA separately for the relative levels of the eight classes of lipid (mol%) (Fig. 2b). The results of the PCA of lipid composition differed from those for lipid content (Fig. 2a and b). PC1 was represented mainly by PA, PG and PI (Additional file 1: Table S2), and PC2 was represented by DGDG, MGDG and phosphatidylserine (PS). This analysis also indicated a clear separation between *Arabidopsis* samples subject to freezing-induced lethal treatment and all other samples (PC1). Along PC2, dead samples and live samples were clearly separated.

**Fig. 3:**
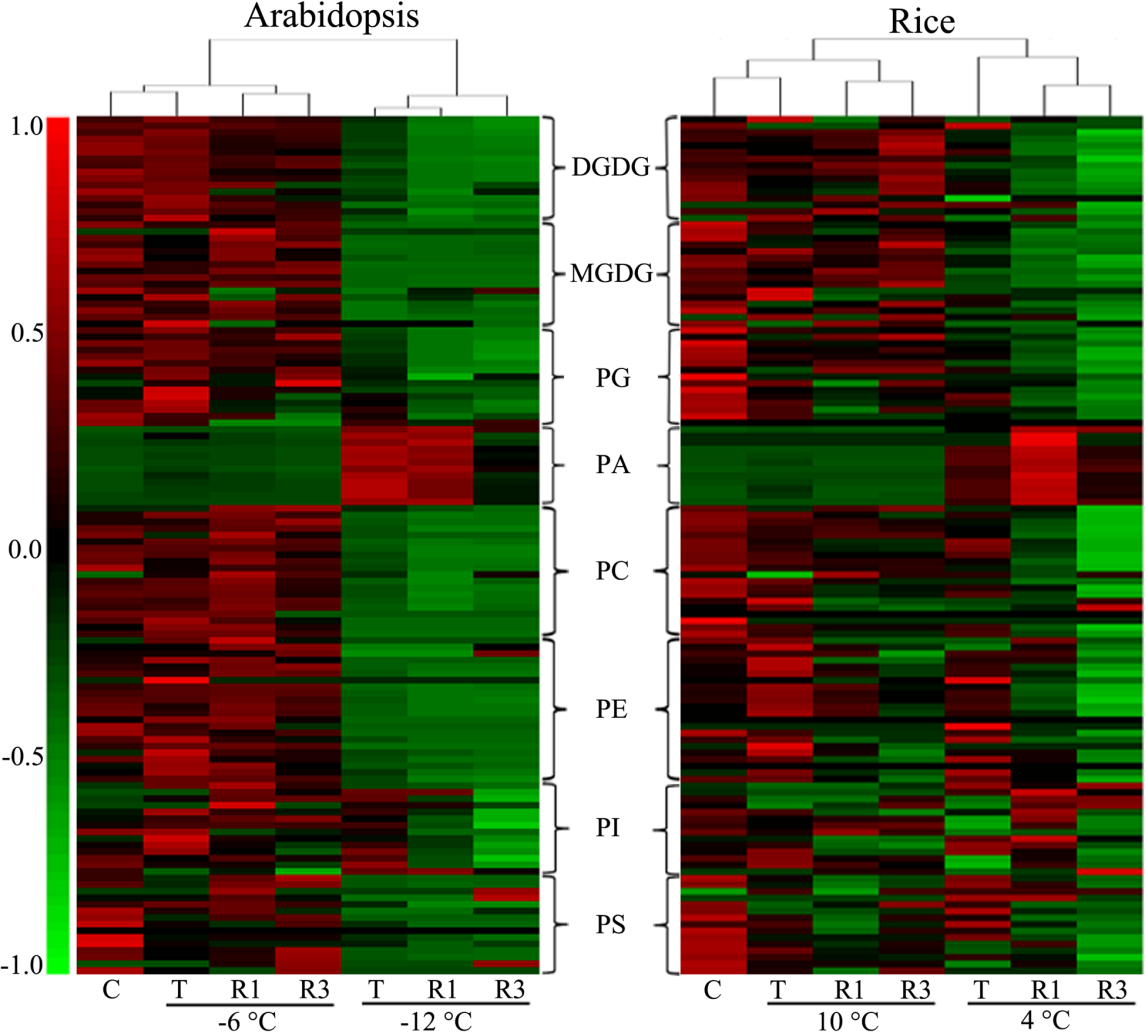
Hierarchical average linkage clustering of lipid molecular species contents (nmol per mg dry weight). *Left panel*: *Arabidopsis* treated at sub-zero temperatures with subsequent recovery culture for 1 and 3 days. *Right panel*: Rice treated at low temperatures above 0 °C with subsequent recovery culture for 1 and 3 days. Each coloured bar within a column represents a lipid molecular species in the indicated plants and treatments. The mean value of the same lipid molecular species in different plants and treatments in a row was calculated. The row-wise mean is subtracted from the values in each row of data, so that the mean value of each row is zero. Each coloured bar within a row represents the relative changes from the mean centre of each lipid species. A total of 130 lipid species in the indicated lipid classes were organised using class (as indicated), total acyl carbons (in ascending order within a class) and total double bonds (in ascending order with class and total acyl carbons). “C” represents “control”, “T” is samples taken immediately after treatment, and “R1” and “R3” indicate samples collected after recovery culture for 1 and 3 days, respectively

To appreciate the overall effects of freezing and chilling injury and subsequent recovery, hierarchical clustering of the lipid profiles was applied (Fig. 3). With respect to the non-lethal low-temperature treatment, the lipid profiles of the samples after treatment were similar to those of the control, except for PI, which increased after treatment in *Arabidopsis* (Fig. 3 Columns C and T (6 °C)) and rice (Columns C and T (10 °C)). Substantial lipid degradation did not occur in *Arabidopsis* during the period of recovery culture. However, in rice, marked phospholipid degradation occurred during the recovery growth period. In the treatments at lethal low temperatures, freezing-induced lipid degradation in *Arabidopsis* was more severe than chilling-induced lipid degradation in rice. In addition, in rice, the levels of some molecules of PE and PS even increased after treatment (Fig. 3, Columns C and T (4 °C)).

### Lipid degradation during low-temperature treatment and subsequent recovery culture in *Arabidopsis* and rice

**Fig. 4:**
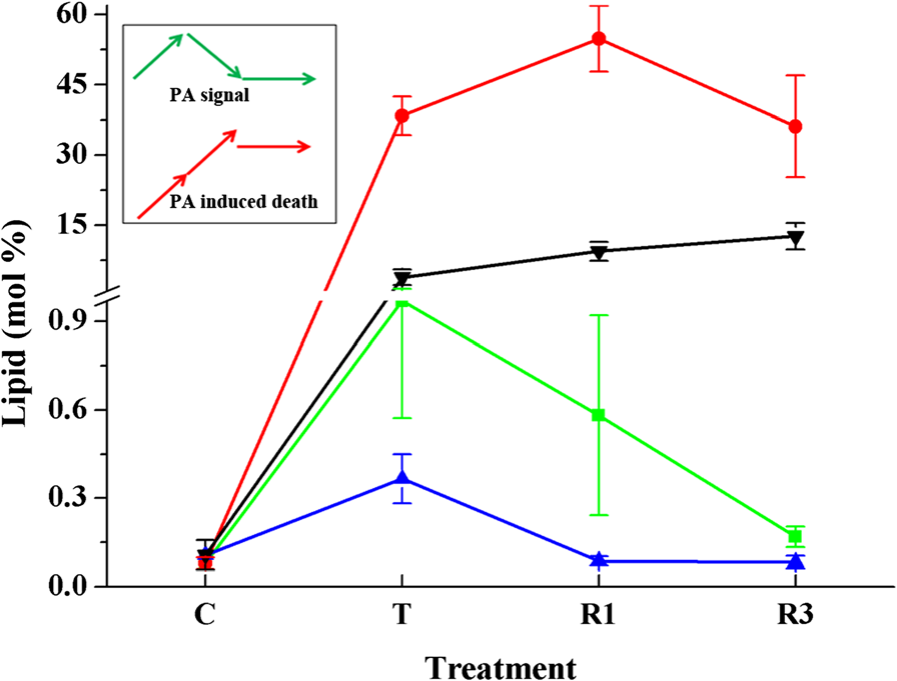
Relative content (mol %) of PA in leaves of *Arabidopsis* and rice after various low-temperature treatments. Inset, models of PA change during non-lethal (*green arrow*) and lethal (*red arrow*) low temperature treatment. *Green line*, *Arabidopsis* treated at non-lethal low temperature. *Blue line*, rice treated at non-lethal low temperature. *Red line*, *Arabidopsis* treated at lethal low temperature. *Black line*, rice treated at lethal low temperature. “C” represents “control”, “T” is sample taken immediately after treatment, and “R1”and “R3” indicate samples collected after recovery culture for 1 and 3 days, respectively. Values are means ± S.D. (*n* = 4 or 5)

After non-lethal low-temperature treatment (T), the levels of total lipids decreased to 78 % and 80 % of the control (C) levels in *Arabidopsis* and rice, respectively (Table 1 and Additional file 1: Table S3). These decreases in total lipids were due mostly to the degradation of MGDG, which was the most abundant of the lipids tested (Table 1). In *Arabidopsis*, PS was the group of lipids that decreased the most, by more than 50 %. In contrast, in rice, PG decreased by 44 %, which was the largest drop. The level of DGDG rose after treatment at a non-lethal low temperature in both plants. PA levels, which can be increased by low temperature stress and as a degradation product, reached 9.25 and 2.71-times the control levels after treatment of *Arabidopsis* and rice, respectively (Fig. 4). After recovery culture for 3 days, the total lipids in *Arabidopsis* rose to 93 % of the control level, and this value was 91 % in rice. The level of most glycerolipids, except for PG, reached at least 90 % of the control levels in *Arabidopsis*. However, in rice, most lipids, including PG, PA, PC and PC only reached about 70 % of the control levels. PG recovered the slowest; after 3 days of recovery culture, its levels were 87 % and 56 % in *Arabidopsis* and rice, respectively (Table 1 and Additional file 1: Table S3).

Treatment at lethal low temperatures induced severe lipid degradation in both *Arabidopsis* and rice. The content of total lipids in *Arabidopsis* decreased by 80 %, while it decreased by 40 % in rice (Table 1). However, the levels of some lipids increased after treatment. The content of PI increased in *Arabidopsis* and that of PE increased in rice. PA in *Arabidopsis* rose to about 100- times its level in the control and its level in rice rose to about 20-times the control level (Fig. 4). The severe degradation of glycerolipids and the considerable increase in PA level in *Arabidopsis* after lethal freezing treatment suggest that the rise of PA was due in part to the degradation of other glycerolipids.

### Lipid profile changes during low-temperature treatment of *Arabidopsis* and rice

**Fig. 5:**
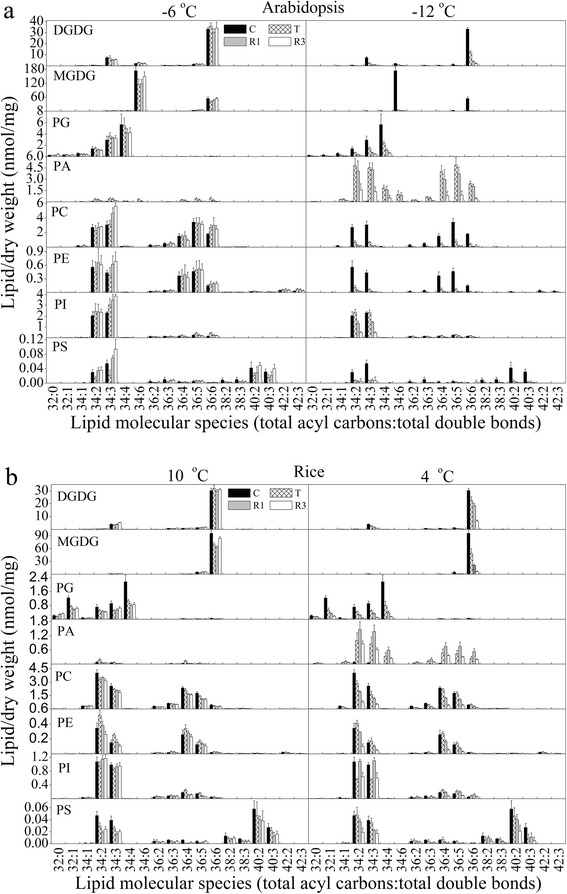
Changes in lipid molecular species during various cold treatments and subsequent recovery culture of *Arabidopsis* (**a**) and rice (**b**). **a** *Left panel*: galactolipids and phospholipids of *Arabidopsis* after −6 °C treatment; *right panel*, galactolipids and phospholipids of *Arabidopsis* after −12 °C treatment. **b** *Left panel*: galactolipids and phospholipids of rice after 10 °C treatment; *right panel*, galactolipids and phospholipids of rice after 4 °C treatment. The dry weight is the dry weight minus lipids (i.e., the dry weight after lipid extraction). “C” means control, “T” indicates samples taken immediately after each cold treatment, and “R1” and “R3” indicate samples taken after posttreatment recovery culture for 1 and 3 days, respectively. Values are means ± S.D. (*n* = 5)

**Fig. 6:**
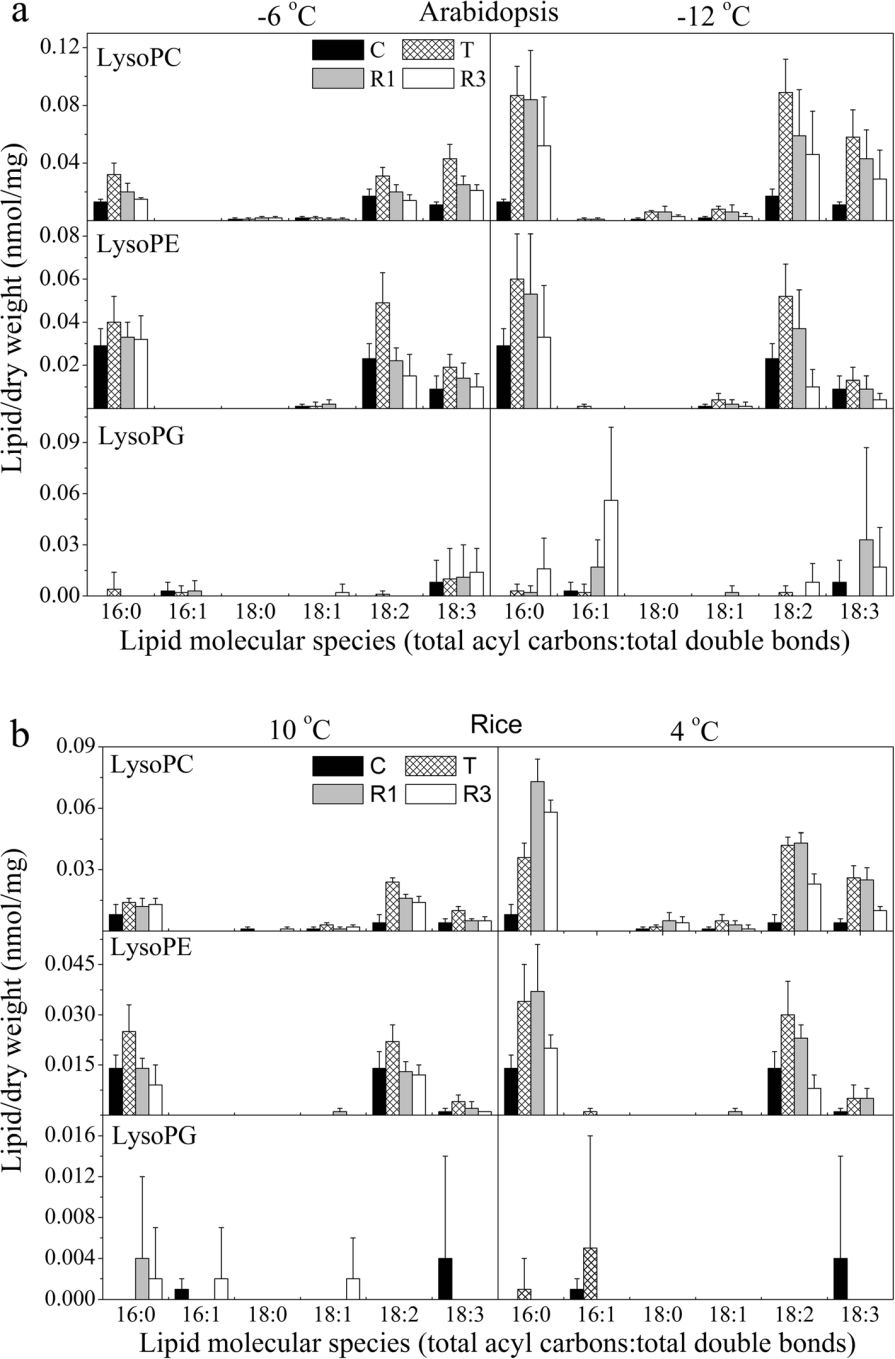
Changes in lysophospholipid molecular species during various cold treatments and subsequent recovery culture of *Arabidopsis* (**a**) and rice (**b**). **a** *Left panel*: galactolipids and phospholipids of *Arabidopsis* after −6 °C treatment; *right panel*: galactolipids and phospholipids of *Arabidopsis* after −12 °C treatment. **b** *Left panel*: galactolipids and phospholipids of rice after 10 °C treatment; *right panel*, galactolipids and phospholipids of rice after 4 °C treatment. The dry weight is the dry weight minus lipids (i.e., the dry weight after lipid extraction). “C” means control, “T” indicates samples taken immediately after each cold treatment, and “R1” and “R3” indicate samples taken after posttreatment recovery culture for 1 and 3 days, respectively. Values are means ± S.D. (*n* = 5)

Detailed analyses of the lipid profiles indicated that the changes in phospholipids, galactolipids and lysophospholipids in the two plants were similar during non-lethal low-temperature treatment (Figs. 5 and 6). There were, however, minor differences between the two plants in PG species 32:1, 34:3 and 34:4; PC species 34:3 and 34:6; 34:3 PI; 34:3 PS; and lysoPG species 16:0 and 18:3. The trends in the responses of 36:6 MGDG and 36:6 DGDG to non-lethal treatment were similar in *Arabidopsis* and rice, while the change in 36:6 MGDG differed from that of 36:6 DGDG during treatment. The level of 36:6 DGDG rose after treatment and reached the level of the control after 3 days of recovery culture. However, the level of 36:6 MGDG decreased after cold treatment then rose to the level of the control after recovery culture (Fig. 5; Additional file 1: Table S1). The 34:1 PG content did not show any marked changes, whereas the level of 34:4 PG decreased dramatically, especially in rice (Fig. 5; Additional file 1: Table S4).

The trends in the response of the glycerolipidome to lethal low-temperature treatment were similar in *Arabidopsis* and rice (Figs. 5 and 6). Interestingly, during such treatments, we detected some PA molecules that could not be detected in the non-lethal treatment, such as 32:0, 36:2 and 36:3 PA in both species, and 34:5 PA in *Arabidopsis* (Fig. 5; Additional file 1: Table S5). Low-temperature treatment could induce the generation of 34:6 PA in *Arabidopsis*, but we could not detect this lipoid molecule at all in rice. We hypothesize that 34:6 PA in *Arabidopsis* is generated by the degradation of 34:6 MGDG. In *Arabidopsis*, the trends of changes of PI species 34:2, 34:3 and 36:5 were similar to those of the corresponding lipid species of PA. It was shown that 34:2 and 34:3 PI are the most abundant PI species in *Arabidopsis*; they increased after freezing and decreased dramatically thereafter during the recovery culture period. Meanwhile, in rice, the levels of these two PI species decreased after chilling and then increased considerably after 1 day of recovery culture (Fig. 5). Most of the lysoPC and lysoPE species increased dramatically in both plants during low-temperature treatment (Fig. 6). This might indicate that the degradation of PC and PE might be carried out partly by phospholipase A during low-temperature-induced death.

### Total levels of ACL and unsaturation of membrane glycerolipids were maintained during low-temperature treatment in both *Arabidopsis* and rice

We used ACL and DBI to represent the fluidity of membranes. A low ACL and a high DBI increase the fluidity of a membrane. The degree of ACL of most lipid classes and of total lipids was maintained during non-lethal and lethal low-temperature treatments, except for that of PA and PS (Table 2). The ACL of PA increased in both plants, which might indicate that lipids with long fatty acid chains were degraded to PA. After recovery culture for 1 day following non-lethal low-temperature treatment, the ACL of PS decreased by almost one carbon in *Arabidopsis*, while it increased by almost 0.5 carbons in rice. The ACL of PS in both plants decreased during lethal low-temperature treatment (Table 2).

A high DBI indicates the presence of more highly unsaturated membrane lipids, and vice versa [15]. During non-lethal low-temperature treatment, the DBI of most glycerolipids was maintained, except for PA and PG (Table 3). The DBI of PG decreased in rice and was maintained in *Arabidopsis*. The DBI of PA in both plants increased after treatment and during the subsequent recovery culture period, but this increase was much greater in *Arabidopsis* than in rice (Table 3). During lethal low-temperature treatment, the DBI of total membrane lipids of *Arabidopsis* decreased dramatically immediately after treatment, and decreased further during the subsequent recovery culture process. This decrease was due mainly to the decrease of the DBI of the most abundant lipids, such as PE (Tables 1 and 3). After 3 days of recovery culture, compared to the control level, the DBI of most lipids decreased, except for PA. The DBI of the total membrane lipids also decreased dramatically in rice. These results indicate that the degree of unsaturation of membrane glycerolipids was maintained in *Arabidopsis* and rice during non-lethal low-temperature treatment, and that the lipid degradation induced by lethal low-temperature treatments might have involved the degradation of lipids with more double bonds first.

## Discussion

The responses of plants to low temperatures, especially their acclimation to cold, have been investigated extensively. Transcriptional factors, such as CBF, play important roles in *Arabidopsis* and rice responses to low temperatures [29]. Membranes are the initial temperature sensors, and remodelling of membrane lipids is one of the most important mechanisms used by plants to respond to low temperatures [13, 18]. Low temperature could induce the decrease of sterols in chilling sensitive plants, for example *Vigna angularis* [30]. Few studies have used lipidomic approaches to investigate the different influences of plant injuries induced by low temperatures below 0 °C (freezing) and above 0 °C (chilling) on cellular glycerolipid profiles on a large scale [12, 22]. Most of these previous studies exclusively used *Arabidopsis*, a species that can tolerate sub-zero low temperatures [13, 19], as the test plant. Some plants, such as rice and cucumber, cannot even endure low temperatures above freezing (4 °C), but little is known about how their lipid profiles respond to chilling-induced plant injury. In the study reported herein, we used rice and *Arabidopsis* to investigate the lipidomic response to chilling- and freezing-induced cell injury.

16:3 and 18:3 plants have different lipid synthesis pathways. It has been shown that 16:3 and 18:3 plants have similar lipid and fatty acid compositions, except for 34:6 MGDG, which is not present in the latter group [15]. Li et al. found that the prokaryotic-type lipid synthesis pathway was upregulated by low temperature and the eukaryotic-type pathway was promoted by high temperature in *Arabidopsis* [31]. Rice is an 18:3 plant, which is chilling-sensitive, while *Arabidopsis* is a freezing-tolerant 16:3 plant. Against this background, the following question arises: do the different pathways for lipid synthesis contribute to the differences between *Arabidopsis* and rice in their capacity to tolerate low temperatures? The content of polyunsaturated lipids and the degree of unsaturation of lipids are two important factors that affect plant tolerance of low temperatures [15, 32]. The particular lipid synthesis pathway used by a plant does not affect its trienoic acid content or its degree of lipid unsaturation [15, 33, 34]. *Solanum nodiflorum* and *Spinacia oleracea* are 16:3 plants that are cold-sensitive, whereas *Saussurea medusa,* which is an 18:3 plant, can tolerate sub-zero low temperatures in alpine screes [15]. Thus, the differences in lipid synthesis between *Arabidopsis* and rice might not contribute to the differences in these two plants' capacities to tolerate low temperatures.

The ACL and DBI of membrane glycerolipids are two important factors that determine the fluidity of membranes. Fatty acids with longer chains and a lower DBI can make the membrane environment more gel-like [15, 35]. It has been reported that the ACL decreases in low-temperature grown bacteria [36] and the DBI tends to increase in plants at low temperatures [37]. In this work, the ACL of the total lipids of cold acclimated *Arabidopsis* was shown to be one carbon less than that in normal growth rice, and the levels of DBI of most phospholipids and MGDG of *Arabidopsis* were higher than in rice. This lower ACL and higher DBI of *Arabidopsis* may give its membranes more fluidity under low-temperature stress, which makes this plant more tolerant to low-temperature. However, the ACL and DBI changed little during low-temperature treatment in either plant. Time and energy are required to change the ACL and DBI. For example, the synthesis of fatty acids from acetyl-CoA and malonyl-CoA requires at least 30 enzymatic reactions [38]. Alteration of the degree of saturation of glycerolipids requires more energy than glycerolipid turnover and is a response of plants to long-term extreme temperatures [15]. In light of this, we conjecture that adjustments of the levels of ACL are also long-term adaptations by plants to extreme temperatures.

Fatty acids with chain lengths of 16 and 18 carbons are the major fatty acids in plants [38]. Most glycerolipids are first synthesized using only palmitic acid (16:0) and oleic acid (18:1), and acyltransferases, including those involved in glycerolipid synthesis, preferentially incorporate these two fatty acids [39, 40]. Glycerolipids that contain 34 and 36 acyl carbons were the most common groups among the lipids determined in our lipidomic investigation, and glycerolipids containing VLCFAs were less abundant. VLCFAs are synthesized in the endoplasmic reticulum (ER) through fatty acid elongase (FAE) complexes starting from 18-carbon chains [28], and *FAE* is not expressed in vegetative tissues [41]. Burgos et al. considered that glycerolipids with VLCFAs are almost exclusively extrachloroplastic [42]. However, we found some chloroplastic glycerolipids with VLCFAs and the relative content of these lipids was almost double that of extrachloroplastic VLCFA-containing glycerolipids. Overexpression of the *FAE1* gene could induce the accumulation of VLCFAs in the galactolipids of chloroplast membranes [43]. The synthesis of galactolipids with VLCFAs may occur either through VLCFA transport from the ER to chloroplast membranes, or through glycerolipid turnover instead of via a *de novo* synthesis pathways in chloroplast membranes. In a previous study, we found that, among eight glycerolipid classes, the ACL of PS was the highest and showed high diversity in different plant species, and that it increased during plant development and under environmental stresses [44]. Millar et al. considered that the accumulation of VLCFAs is deleterious to cells because they can perturb the integrity of membrane structures [45]. The ACL of PS increased in both *Arabidopsis* and rice during non-lethal low-temperature treatment, and then decreased rapidly in *Arabidopsis* after treatment. The capacity to remove very long fatty acids of PS in *Arabidopsis* plants under low-temperature stress may contribute to their greater low-temperature tolerance than rice.

PG is the major phospholipid in chloroplast membranes, and the composition of PG molecular species is correlated closely with the sensitivity of plants to low temperatures. Plants that have more PG molecules with a high melting point, such as 16:0/16:0 (32:0) and 16:0/16:1(3 *t*) (32:1), are sensitive to low-temperature stresses [26, 27]. In our previous study, we found that the level of high-melting-point PG molecules in alpine plants was very low [15]. The abundance of high-melting-point PG molecules in rice chloroplast membranes might make these membranes more gel-like, preventing tolerance of low-temperature stresses at above 0 °C. This could well be one important factor that contributes to the difference between *Arabidopsis* and rice in the capacity to tolerate low temperature.

Galactolipids, such as MGDG and DGDG, are the main components of chloroplast membranes, which play an important role in photosynthesis [46]. The ratio of MGDG to DGDG is crucial for the physical phase of thylakoid membranes and might be adjusted as a response to temperature stresses. DGDG has relatively large head groups, which have the propensity to form bilayer membranes, and the transformation of MGDG into DGDG has been shown to be one of the most important mechanisms by which plants respond to freezing stresses [18]. An *Arabidopsis* mutant with a high ratio of MGDG/DGDG was more sensitive to heat stresses [47]. Surprisingly, even though the capacity of *Arabidopsis* to tolerate low temperatures was greater than that of rice, the ratio of MGDG/DGDG in *Arabidopsis* was far higher than that in rice. Mishra found that the MGDG/DGDG ratio was correlated with the stage of development of plants [48]. However, the decrease of the ratio of MGDG/DGDG in *Arabidopsis* was higher than that in rice after non-lethal low-temperature treatment. We speculate that the MGDG/DGDG ratio may not be a determinant of low-temperature tolerance between different plant species, but that the capacity of plants to regulate their MGDG/DGDG ratio may be positively correlated with tolerance of low temperatures.

PI is the third most abundant extraplastidic lipid. It is a precursor for inositol-containing lipids. PI and its derivatives, polyphosphoinositides, play important roles in stress responses [49–51]. In our experiments, the DBI and ACL of PI were found to be highly conserved, with no differences between *Arabidopsis* and rice during treatment. Most glycerolipids were degraded after low-temperature treatment, but the content of PI in *Arabidopsis* increased by about 50 % after freezing. However, it remained unchanged in rice after chilling treatment. Liu found that the overexpression of PI synthase could improve drought tolerance in maize [52]. Both freezing and drought can induce osmotic stress in plants, so PI or its derivatives might participate in plant responses to freezing-induced osmotic stress. The capacity to synthesise PI at low temperatures may correlate with the sensitivity of plants to low temperatures. Little is known about signals transduced by PI and its derivatives under freezing stresses, so further studies should be performed using mutants to investigate the relationship between the level of PI and the capacity of plants to tolerate low temperatures.

PA is a very important glycerolipid. It is an intermediate in glycerolipid synthesis and degradation [36, 53]. It also acts as a signalling lipid that participates extensively in the closure of stomata and in stress responses in plants [24, 54–56]. However, the accumulation of PA can compromise membrane integrity because it has a cone-like geometry and a tendency to form the hexagonal II phase of membranes [16, 19]. Low temperature induced an increase of PA in both *Arabidopsis* and rice. Immediately after lethal low-temperature treatment, PA reached 98 and 20-times the control levels in *Arabidopsis* and rice, respectively, which was far higher than the increase induced by non-lethal low temperatures. The non-lethal low temperature-induced rise in PA could be countered in the recovery culture period. However, the increased PA pool induced by lethal low temperatures was too great for the plants to remove, resulting in a very large PA pool in cells.

We speculate that the ability to remove the low-temperature-induced PA pool is related closely to plants’ capacity to tolerate low temperatures. The accumulation of PA could lead to lesions and loss of the osmoregulatory capacity of membranes; finally, water would flood into cells, causing them to burst. Low-temperature-induced PA accumulation in plant cells may thus be the main reason for cell damage. PA may play dual roles in both chilling and freezing stresses (Fig. 4): during non-lethal low temperature treatment, PA may act as a signal; its level increased greatly immediately after treatment, then decreased to the level of the control during the recovery process. However, on lethal low temperature treatment, PA increased significantly and was maintained at a very high level, inducing cell death.

## Conclusions

This paper describes the response of glycerolipidome to chilling and freezing induced injuries in chilling-sensitive plant rice and freezing-tolerant plant *Arabidopsis* respectively. The two plants have different lipid synthesis pathways, which rice is an 18:3 plant and *Arabidopsis* is a 16:3 plant, however, this difference between the two plants may not correlate to their different low temperature tolerant ability. *Arabidopsis* has higher levels of galactolipids and DBI, and lower levels of ACL than rice, which may be one important reason for its more low temperature tolerance ability. The speed of removing VLCFAs of PS in *Arabidopsis* is quicker than that in rice, which could reduce the VLCFAs caused injuries in cells. The decrease of the rate of MDGD/DGDG is higher in *Arabidopsis* than that in rice, and this may contribute to maintain the integrity of chloroplast membranes during low temperature stress. The ability of plants to regulate the ratio of MGDG/DGDG may be one important determinant to low temperature tolerant ability in plants. Low temperature induces the rise of PA, and PA may play dual roles during low temperature stress, which as a signal lipid helping plants tolerate to low temperatures, or as a structural molecule leading to lesions in membranes and finally causing the cell death.

## Methods

### Plant materials and treatments

Seeds of *Arabidopsis* (*Arabidopsis thaliana*) were of the Wassilewskija ecotype and collected from plants bred in our experimental lab. Rice (*Oryza sativa* indica) seeds were purchased from Jingdian Seed Company (Kunming, China). For *Arabidopsis*, seeds were cold-stratified at 4 °C for 3 days before culture. All plants were grown in soil (humus soil:clay 4:1) and watered every week; one-quarter Hoagland solution was added to soil every month. The normal growth conditions were 22 °C (day) and 18 °C (night) with fluorescent lighting of 120 μmol · m-^2^ · s^-1^, 60 % relative humidity, and a 12 h photoperiod. Before freezing, 42-day-old *Arabidopsis* seedlings were first cold-acclimated at 4 °C for 3 days. After this cold acclimation, the seedlings were subjected to a temperature drop from 4 to −6 °C at 2 °C/h in the growth chamber, which was maintained at −6 °C for 2 h. Then the temperature was raised to 4 °C at 2 °C/h. Another group of cold-acclimated *Arabidopsis* was subjected to −12 °C for 10 h and then kept at 4 °C in a growth chamber. After 12 h of further exposure to 4 °C, all *Arabidopsis* plants were placed in a growth chamber under normal growth conditions for recovery culture for 1 and 3 days. For chilling rice, 21-day-old rice seedlings were placed in a growth chamber at 4 or 10 °C for 6 days, then allowed to recover for 1 and 3 days under normal conditions. Cold-acclimated *Arabidopsis* and rice grown under normal conditions were used as controls, labelled with the letter “C” in the Tables and Figures. Samples harvested after freezing and chilling treatment are labelled “T”. Plants after 1 and 3 days of recovery culture are labelled “R1” and “R3”, respectively.

### Lipid extraction and ESI-MS/MS analysis

Lipid extraction, ESI-MS/MS analysis and quantification were performed as described previously, with minor modifications [19, 25] (Kansas Lipidomics Research Centre, http://www.k-state.edu/lipid/lipidomics). Fully expanded *Arabidopsis* and rice leaves were harvested at the sampling time and, to inhibit lipolytic activity, were transferred immediately into 3 ml of isopropanol with 0.01 % butylated hydroxytoluene at 75 °C. The tissue was extracted three times with chloroform/methanol (2:1), with 12 h of agitation each time. The remaining plant tissue was dried overnight at 105 °C and weighed to give the dry weight of the plants. Lipid samples were analysed on a triple quadrupole MS/MS equipped for ESI. Data were processed as described previously [19, 25]. The lipids in each head-group class were quantified in comparison to two internal standards for the class. Five replicates of each treatment were analysed for each plant.

### Statistical procedures

The Q-test was performed on the total amount of lipid in each head-group class and data from discordant samples were removed [19, 25]. The data were subjected to one-way analysis of variance (ANOVA) with SPSS 13.0. Statistical significance was tested by Fisher’s least significant difference (LSD) method. Hierarchical clustering analysis was performed using Cluster 3.0 and Java TreeView. Principal component analysis was conducted with SPSS 13.0. DBI and ACL were calculated as described previously [14]: DBI = (∑[*N* × mol % lipid])/100, where *N* is the number of double bonds in each lipid molecule; ACL = (∑[n × mol % lipid])/100, where n is the number of acyl carbons in each lipid molecule.

### Availability of supporting data

The datasets supporting the results of this article are included within the article and its additional files.

## Additional file

Additional file 1: Figure S1. Hierarchical average linkage clustering of lipid molecular species relative contents (mol%). **Table S1.** Levels of galactolipid species in leaves of *Arabidopsis* and rice after various low-temperature treatments. **Table S2.** Component matrix of PCA analysis. **Table S3.** Lipid ratios of *Arabidopsis* and rice after various low-temperature treatments. **Table S4.** Levels of PG molecular species in leaves of *Arabidopsis* and rice after various low-temperature treatments. **Table S5.** Levels of PA molecular species in leaves of *Arabidopsis* and rice after various low-temperature treatments. (DOC 446 kb)

## Abbreviations

ACL: acyl chain length
DBI: double bond index
DGDG: digalactosyldiacylglycerol
MDGD: monogalactosyldiacylglycerol
PA: phosphatidic acid
PC: phosphatidylcholine
PE: phosphatidylethanolamine
PG: phosphatidylglycerol
PI: phosphatidylinositol
PS: phosphatidylserine
