# Supplementary material for: Glycerolipidome responses to freezing- and chilling-induced injuries: examples in Arabidopsis and rice
Source: BMC Plant Biol. 2016 Mar 22;16:70. doi: 10.1186/s12870-016-0758-8 (PMC4802656; doi:10.1186/s12870-016-0758-8)
Supplement: Additional file 1: Figure S1. — Hierarchical average linkage clustering of lipid molecular species relative contents (mol%). Table S1. Levels of galactolipid species in leaves of Arabidopsis and rice after various low-temperature treatments. Table S2. Component matrix of PCA analysis. Table S3. Lipid ratios of Arabidopsis and rice after various low-temperature treatments. Table S4. Levels of PG molecular species in leaves of Arabidopsis and rice after various low-temperature treatments. Table S5. Levels of PA molecular species in leaves of Arabidopsis and rice after various low-temperature treatments. (DOC 446 kb) [file 12870_2016_758_MOESM1_ESM.doc]

**Differential glycerolipidome remodelling between chilling- and freezing-induced injuries: examples in rice and *Arabidopsis***

Guowei Zheng^1,2,#^, Lixia Li^3,#^, Weiqi Li^1,2*^

^1^Key Laboratory for Plant Diversity and Biogeography of East Asia, Kunming Institute of Botany, Chinese Academy of Sciences, Kunming, Yunnan 650202, People’s Republic of China

^2^Germplasm Bank of Wild Species, Kunming Institute of Botany, Chinese Academy of Sciences, Kunming, 650201, People’s Republic of China

^3^Guiyang medicinal botanical garden, Guiyang, 550002, People’s Republic of China

^#^These authors contributed equally to this work.

^*^Corresponding author (E-mail: [weiqili@mail.kib.ac.cn](mailto:weiqili@mail.kib.ac.cn))

Supplementary Materials


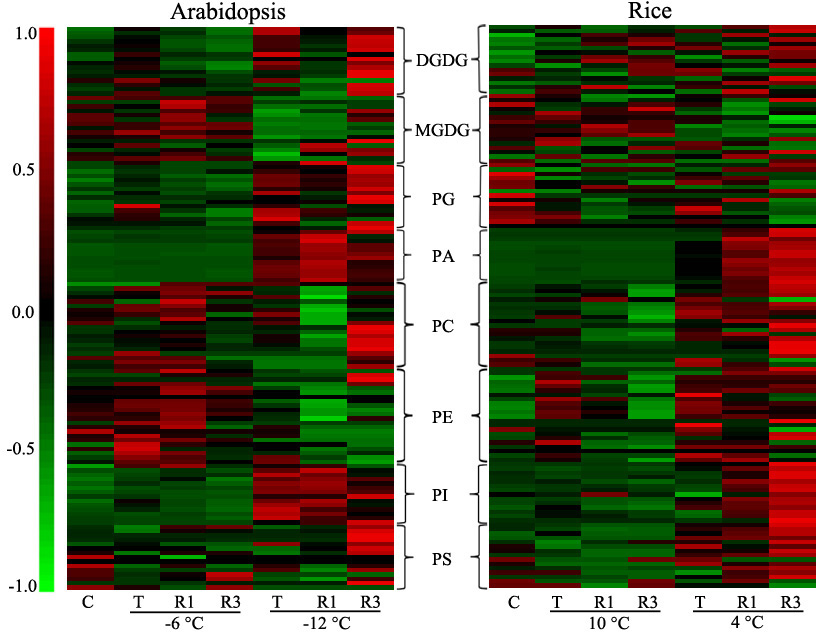


**Fig. S1.** Hierarchical average linkage clustering of lipid molecular species relative contents (mol%). Left panel: *Arabidopsis* treated at sub-zero temperatures with subsequent recovery culture for 1 and 3 days; right panel: rice treated at above-zero low temperatures with subsequent recovery culture for 1 and 3 days. Each coloured bar within a column represents a lipid molecular species in the indicated plants and treatments. The mean value of the same lipid molecular species in different plants and treatments in a row was calculated. The row-wise mean is subtracted from the values in each row of data, so that the mean value of each row is zero. Each coloured bar within a row represents the relative changes from the mean centre of each lipid species. A total of 130 lipid species in the indicated lipid classes were organised using class (as indicated), total acyl carbons (in ascending order within a class) and total double bonds (in ascending order with class and total acyl carbons). “C” represents “control”, “T” indicates samples taken immediately after each cold treatment, and “R1”and “R3” indicate samples after posttreatment recovery culture for 1 and 3 days, respectively.

## Table S1. Levels of galactolipid species in leaves of *Arabidopsis* and rice after various low-temperature treatments. “C” represents “control”, “T” indicates samples taken immediately after each cold treatment, and “R1”and “R3” indicate samples taken after post treatment recovery culture for 1 and 3 days, respectively. Values are means ± S.D. (n=4 or 5).

|  | **Plant species** |  |  |  |  | |  |  |  |  |  |
| --- | --- | --- | --- | --- | --- | --- | --- | --- | --- | --- | --- |
| **Lipid species** |  |  |  | *Arabidopsis* (-6 °C)/Rice (10 °C) | | | |  | *Arabidopsis* (-12 °C)/Rice (4 °C) | | |
|  |  | C |  | T | R1 | | R3 |  | T | R1 | R3 |
|  |  |  |  |  |  |  |  |  |  |  |  |
|  |  | Lipid content (nmol per mg dry weight) | | | | | | | | | |
| 34:6 DGDG | *Arabidopsis* | 2.20 ± 0.13 |  | 2.94 ± 0.35 | | 2.25 ± 0.27 | 2.07 ± 0.51 |  | 1.18 ± 0.13 | 0.42 ± 0.03 | 0.22 ± 0.09 |
|  | Rice | 0.01 ± 0.00 |  | 0.01 ± 0.01 | | 0.00 ± 0.00 | 0.01 ± 0.00 |  | 0.00 ± 0.00 | 0.01 ± 0.00 | 0.00 ± 0.00 |
|  |  |  |  |  | |  |  |  |  |  |  |
| 34:6 MGDG | *Arabidopsis* | 174.00 ± 16.46 |  | 114.81 ± 23.44 | | 117.91 ± 18.46 | 148.87 ± 20.38 |  | 5.08 ± 0.80 | 2.18 ± 0.36 | 1.59 ± 0.35 |
|  | Rice | 0.06 ± 0.01 |  | 0.04 ± 0.01 | | 0.02 ± 0.01 | 0.03 ± 0.01 |  | 0.03 ± 0.01 | 0.02 ± 0.01 | 0.01 ± 0.00 |
|  |  |  |  |  | |  |  |  |  |  |  |
| 36:6 DGDG | *Arabidopsis* | 33.31 ± 1.41 |  | 35.83 ± 3.16 | | 30.37 ± 2.95 | 34.12 ± 5.53 |  | 12.12 ± 1.53 | 3.95 ± 0.65 | 1.99 ± 0.66 |
|  | Rice | 30.31 ± 1.72 |  | 31.97 ± 2.52 | | 29.61 ± 1.59 | 30.89 ± 1.03 |  | 22.63 ± 2.83 | 18.60 ± 1.69 | 6.48 ± 0.94 |
|  |  |  |  |  | |  |  |  |  |  |  |
| 36:6 MGDG | *Arabidopsis* | 54.12 ± 6.61 |  | 38.42 ± 5.96 | | 45.80 ± 4.91 | 54.53 ± 4.71 |  | 2.26 ± 0.27 | 1.41 ± 0.30 | 0.90 ±0.25 |
|  | Rice | 95.84 ± 6.42 |  | 68.60 ± 4.70 | | 62.72 ± 3.61 | 83.93 ± 4.35 |  | 48.81 ± 10.86 | 20.83 ± 2.37 | 5.66 ± 2.10 |
|  |  |  |  |  | |  |  |  |  |  |  |

**Table S2.** Component matrix of PCA analysis.

|  | nmol | |  | mol% | |
| --- | --- | --- | --- | --- | --- |
|  | Component | |  | Component | |
|  | 1 | 2 |  | 1 | 2 |
| DGDG | .903 | -.155 |  | .311 | .698 |
| MGDG | .960 | .053 |  | .719 | -.679 |
| PG | .855 | .416 |  | -.876 | .001 |
| PA | -.576 | .741 |  | -.900 | .333 |
| PC | .964 | -.135 |  | .731 | .631 |
| PE | .934 | .112 |  | .750 | .332 |
| PI | .697 | .684 |  | -.789 | .544 |
| PS | .783 | -.371 |  | .360 | .804 |

**Table S3**. Lipid ratios of *Arabidopsis* and rice after various low-temperature treatments. The calculation of lipid ratios involved dividing (i) the content of lipid following each treatment by (ii) the content of the control. “T” indicates samples taken immediately after each cold treatment, and “R1”and “R3” indicate samples taken after post treatment recovery culture for 1 and 3 days, respectively.

| Lipid class | Plant species |  | *Arabidopsis* (-6 °C)/Rice (10 °C) | | | |  | *Arabidopsis* (-12°C)/Rice (4°C) | | |
| --- | --- | --- | --- | --- | --- | --- | --- | --- | --- | --- |
|  |  |  |  |  |  |  |  |  |  |  |
|  |  |  | T | R1 | R3 | |  | T | R1 | R3 |
|  |  |  |  |  |  |  |  |  |  |  |
|  |  |  | Lipid ratio divided by control | | | | | | | |
| DGDG | *Arabidopsis* |  | 1.06 ± 0.10 | 0.87 ± 0.08 | | 0.95 ± 0.15 |  | 0.36 ± 0.04 | 0.12 ± 0.02 | 0.07 ± 0.03 |
|  | Rice |  | 1.01 ± 0.08 | 0.99 ± 0.06 | | 1.07 ± 0.03 |  | 0.72 ± 0.09 | 0.58 ± 0.04 | 0.22 ± 0.03 |
|  |  |  |  |  | |  |  |  |  |  |
| MGDG | *Arabidopsis* |  | 0.68 ± 0.11 | 0.74 ± 0.10 | | 0.91 ± 0.10 |  | 0.03 ± 0.00 | 0.02 ± 0.00 | 0.01 ± 0.00 |
|  | Rice |  | 0.71 ± 0.05 | 0.69 ± 0.04 | | 0.90 ± 0.04 |  | 0.49 ± 0.11 | 0.22 ± 0.02 | 0.06 ± 0.02 |
|  |  |  |  |  | |  |  |  |  |  |
| PG | *Arabidopsis* |  | 1.06 ± 0.17 | 0.86 ± 0.09 | | 0.87 ± 0.12 |  | 0.45 ± 0.07 | 0.21 ± 0.04 | 0.13 ± 0.02 |
|  | Rice |  | 0.56 ± 0.05 | 0.50 ± 0.05 | | 0.55 ± 0.07 |  | 0.45 ± 0.08 | 0.27 ± 0.03 | 0.10 ± 0.02 |
|  |  |  |  |  | |  |  |  |  |  |
| PA | *Arabidopsis* |  | 9.25 ± 3.32 | 5.61 ± 2.74 | | 1.98 ± 0.41 |  | 98.65 ± 16.47 | 82.70 ± 23.42 | 23.92 ± 12.90 |
|  | Rice |  | 2.71 ± 0.50 | 0.62 ± 0.11 | | 0.71 ± 0.19 |  | 20.38 ± 7.02 | 33.65 ± 7.21 | 16.07 ± 1.52 |
|  |  |  |  |  | |  |  |  |  |  |
| PC | *Arabidopsis* |  | 0.98 ± 0.17 | 1.20 ± 0.13 | | 1.15 ± 0.08 |  | 0.23 ± 0.06 | 0.05 ± 0.04 | 0.05 ± 0.03 |
|  | Rice |  | 0.84 ± 0.06 | 0.77 ± 0.03 | | 0.72 ± 0.06 |  | 0.78 ± 0.10 | 0.50 ± 0.03 | 0.20 ± 0.05 |
|  |  |  |  |  | |  |  |  |  |  |
| PE | *Arabidopsis* |  | 0.99 ± 0.37 | 1.22 ± 0.45 | | 1.15 ± 0.35 |  | 0.17 ± 0.08 | 0.04 ± 0.03 | 0.03 ± 0.03 |
|  | Rice |  | 1.46 ± 0.17 | 1.07 ± 0.15 | | 0.78 ± 0.12 |  | 1.14 ± 0.16 | 0.72 ± 0.06 | 0.25 ± 0.08 |
|  |  |  |  |  | |  |  |  |  |  |
| PI | *Arabidopsis* |  | 1.33 ± 0.30 | 1.32 ± 0.26 | | 1.33 ± 0.11 |  | 1.09 ± 0.11 | 0.63 ± 0.06 | 0.20 ± 0.05 |
|  | Rice |  | 0.94 ± 0.07 | 0.95 ± 0.09 | | 0.95 ± 0.10 |  | 0.66 ± 0.02 | 1.09 ± 0.09 | 0.60 ± 0.12 |
|  |  |  |  |  | |  |  |  |  |  |
| PS | *Arabidopsis* |  | 0.44 ± 0.14 | 0.87 ± 0.21 | | 1.21 ± 0.21 |  | 0.15 ± 0.04 | 0.09 ± 0.06 | 0.12 ± 0.15 |
|  | Rice |  | 0.69 ± 0.13 | 0.49 ± 0.05 | | 0.59 ± 0.11 |  | 0.79 ± 0.13 | 0.61 ± 0.07 | 0.34 ± 0.13 |
|  |  |  |  |  | |  |  |  |  |  |
| LysoPC | *Arabidopsis* |  | 2.50 ± 0.44 | 1.54 ± 0.34 | | 1.23 ± 0.17 |  | 5.67 ± 1.33 | 4.50 ± 2.11 | 3.03 ± 1.84 |
|  | Rice |  | 2.80 ± 0.26 | 1.88 ± 0.30 | | 1.90 ± 0.39 |  | 6.15 ± 0.67 | 8.22 ± 1.36 | 5.30 ± 0.76 |
|  |  |  |  |  | |  |  |  |  |  |
| LysoPE | *Arabidopsis* |  | 1.77 ± 0.44 | 1.16 ± 0.27 | | 0.93 ± 0.28 |  | 2.10 ± 0.66 | 1.63 ± 0.81 | 0.77 ± 0.46 |
|  | Rice |  | 1.76 ± 0.34 | 1.04 ± 0.10 | | 0.76 ± 0.25 |  | 2.39 ± 0.57 | 2.28 ± 0.64 | 0.97 ± 0.27 |
|  |  |  |  |  | |  |  |  |  |  |
| LysoPG | *Arabidopsis* |  | 1.17 ± 1.63 | 1.52 ± 1.32 | | 1.69 ± 1.55 |  | 0.65 ± 1.16 | 5.05 ± 6.47 | 9.28 ± 3.83 |
|  | Rice |  | 0.00 ± 0.00 | 0.90 ± 1.56 | | 1.36 ± 2.12 |  | 1.20 ± 2.05 | 0.00 ± 0.00 | 0.00 ± 0.00 |
|  |  |  |  |  | |  |  |  |  |  |
| Total | *Arabidopsis* |  | 0.78 ± 0.09 | 0.80 ± 0.09 | | 0.93 ± 0.11 |  | 0.20 ± 0.02 | 0.12 ± 0.02 | 0.05 ± 0.01 |
|  | Rice |  | 0.80 ± 0.05 | 0.76 ± 0.04 | | 0.91 ± 0.04 |  | 0.60 ± 0.09 | 0.38 ± 0.02 | 0.14 ± 0.02 |
|  |  |  |  |  | |  |  |  |  |  |

**Table S4.** Levels of PG molecular species in leaves of *Arabidopsis* and rice after various low-temperature treatments. “C” represents “control”, “T” indicates samples taken immediately after each cold treatment, and “R1”and “R3” indicate samples taken after post treatment recovery culture for 1 and 3 days, respectively. Values are means ± S.D. (n=4 or 5).

| PG species | Plant species |  |  | *Arabidopsis* (-6 °C)/Rice (10 °C) | | |  | *Arabidopsis* (-12°C)/Rice (4°C) | | |
| --- | --- | --- | --- | --- | --- | --- | --- | --- | --- | --- |
|  |  | C |  | T | R1 | R3 |  | T | R1 | R3 |
|  |  | nmol per mg dry weight | | | | | | | | |
|  |  |  | | | | | | | | |
| 32:0 | *Arabidopsis* | 0.213 ± 0.060 |  | 0.257 ± 0.012 | 0.244 ± 0.048 | 0.370 ± 0.113 |  | 0.115 ± 0.044 | 0.052 ± 0.011 | 0.043 ± 0.010 |
|  | Rice | 0.218 ± 0.044 |  | 0.153 ± 0.027 | 0.275 ± 0.037 | 0.324 ± 0.034 |  | 0.138 ± 0.023 | 0.147 ± 0.022 | 0.053 ± 0.019 |
|  |  |  |  |  |  |  |  |  |  |  |
| 32:1 | *Arabidopsis* | 0.279 ± 0.091 |  | 0.331 ± 0.069 | 0.234 ± 0.018 | 0.265 ± 0.062 |  | 0.125 ± 0.028 | 0.075 ± 0.021 | 0.072 ± 0.015 |
|  | Rice | 1.235 ± 0.123 |  | 0.737 ± 0.065 | 0.550 ± 0.052 | 0.620 ± 0.082 |  | 0.525 ± 0.121 | 0.321 ± 0.041 | 0.169 ± 0.042 |
|  |  |  |  |  |  |  |  |  |  |  |
| 34:0 | *Arabidopsis* | 0.050 ± 0.033 |  | 0.068 ± 0.025 | 0.048 ± 0.012 | 0.055 ± 0.035 |  | 0.033 ± 0.012 | 0.009 ± 0.006 | 0.008 ± 0.006 |
|  | Rice | 0.003 ± 0.003 |  | 0.002 ± 0.004 | 0.004 ± 0.006 | 0.004 ± 0.003 |  | 0.003 ± 0.002 | 0.002 ± 0.002 | 0.001 ± 0.001 |
|  |  |  |  |  |  |  |  |  |  |  |
| 34:1 | *Arabidopsis* | 0.559 ± 0.152 |  | 0.410 ± 0.100 | 0.416 ± 0.089 | 0.330 ± 0.139 |  | 0.226 ± 0.090 | 0.113 ± 0.050 | 0.094 ± 0.036 |
|  | Rice | 0.086 ± 0.024 |  | 0.051 ± 0.011 | 0.069 ± 0.019 | 0.068 ± 0.018 |  | 0.027 ± 0.015 | 0.018 ± 0.014 | 0.007 ± 0.008 |
|  |  |  |  |  |  |  |  |  |  |  |
| 34:2 | *Arabidopsis* | 1.380 ± 0.352 |  | 1.458 ± 0.226 | 1.180 ± 0.134 | 1.073 ± 0.138 |  | 0.697 ± 0.043 | 0.320 ± 0.053 | 0.190 ± 0.031 |
|  | Rice | 0.707 ± 0.146 |  | 0.488 ± 0.064 | 0.447 ± 0.056 | 0.420 ± 0.044 |  | 0.428 ± 0.032 | 0.242 ± 0.014 | 0.066 ± 0.014 |
|  |  |  |  |  |  |  |  |  |  |  |
| 34:3 | *Arabidopsis* | 2.922 ± 0.697 |  | 3.625 ± 0.600 | 3.220 ± 0.219 | 3.248 ± 0.464 |  | 1.411 ± 0.278 | 0.641 ± 0.086 | 0.392 ± 0.066 |
|  | Rice | 0.907 ± 0.151 |  | 0.497 ± 0.049 | 0.526 ± 0.077 | 0.648 ± 0.097 |  | 0.481 ± 0.049 | 0.321 ± 0.041 | 0.097 ± 0.023 |
|  |  |  |  |  |  |  |  |  |  |  |
| 34:4 | *Arabidopsis* | 5.644 ± 1.879 |  | 5.449 ± 1.368 | 4.140 ± 0.801 | 4.252 ± 0.838 |  | 2.303 ± 0.358 | 1.133 ± 0.246 | 0.609 ± 0.087 |
|  | Rice | 2.171 ± 0.391 |  | 1.057 ± 0.087 | 0.803 ± 0.086 | 0.843 ± 0.131 |  | 0.800 ± 0.237 | 0.377 ± 0.097 | 0.136 ± 0.030 |
|  |  |  |  |  |  |  |  |  |  |  |
| 36:1 | *Arabidopsis* | 0.003 ± 0.004 |  | 0.002 ± 0.002 | 0.00 ± 0.000 | 0.000 **±** 0.001 |  | 0.002 ± 0.005 | 0.001 ± 0.001 | 0.002 ± 0.005 |
|  | Rice | 0.000 ± 0.000 |  | 0.000 ± 0.000 | 0.000 ± 0.000 | 0.000 ± 0.001 |  | 0.000 ± 0.000 | 0.000 ± 0.000 | 0.000 ± 0.002 |
|  |  |  |  |  |  |  |  |  |  |  |
| 36:2 | *Arabidopsis* | 0.006 ± 0.008 |  | 0.004 ± 0.005 | 0.004 ± 0.003 | 0.000 ± 0.001 |  | 0.003 ± 0.001 | 0.000 ± 0.000 | 0.001 ± 0.001 |
|  | Rice | 0.005 ± 0.006 |  | 0.003 ± 0.002 | 0.001 ± 0.002 | 0.001 ± 0.001 |  | 0.002 ± 0.001 | 0.001 ± 0.001 | 0.000 ± 0.001 |
|  |  |  |  |  |  |  |  |  |  |  |
| 36:3 | *Arabidopsis* | 0.010 ± 0.011 |  | 0.012 ± 0.009 | 0.004 ± 0.006 | 0.002 ± 0.002 |  | 0.007 ± 0.008 | 0.001 ± 0.002 | 0.000 **±** 0.000 |
|  | Rice | 0.003 ± 0.005 |  | 0.002 ± 0.002 | 0.000 ± 0.000 | 0.002 ± 0.002 |  | 0.001 ± 0.002 | 0.001 ± 0.001 | 0.000 ± 0.000 |
|  |  |  |  |  |  |  |  |  |  |  |
| 36:4 | *Arabidopsis* | 0.018 ± 0.013 |  | 0.023 ± 0.019 | 0.009 ± 0.003 | 0.007 ± 0.004 |  | 0.010 ± 0.004 | 0.004 ± 0.003 | 0.001 ± 0.002 |
|  | Rice | 0.031 ± 0.019 |  | 0.022 ± 0.005 | 0.009 ± 0.006 | 0.012 ± 0.008 |  | 0.021 ± 0.013 | 0.004 ± 0.004 | 0.002 ± 0.002 |
|  |  |  |  |  |  |  |  |  |  |  |
| 36:5 | *Arabidopsis* | 0.010 ± 0.016 |  | 0.037 ± 0.017 | 0.015 ± 0.009 | 0.002 ± 0.004 |  | 0.014 ± 0.005 | 0.005 ± 0.004 | 0.001 ± 0.002 |
|  | Rice | 0.027 ± 0.010 |  | 0.016 ± 0.005 | 0.011 ± 0.006 | 0.015 ± 0.008 |  | 0.022 ± 0.006 | 0.007 ± 0.009 | 0.002 ± 0.002 |
|  |  |  |  |  |  |  |  |  |  |  |
| 36:6 | *Arabidopsis* | 0.023 ± 0.017 |  | 0.079 ± 0.034 | 0.035 ± 0.012 | 0.034 ± 0.018 |  | 0.012 ± 0.005 | 0.003 ± 0.002 | 0.002 ± 0.004 |
|  | Rice | 0.061 ± 0.010 |  | 0.024 ± 0.008 | 0.020 ± 0.009 | 0.029 ± 0.012 |  | 0.025 ± 0.015 | 0.011 ± 0.005 | 0.003 ± 0.003 |
|  |  |  |  |  |  |  |  |  |  |  |
| Total 36-C | *Arabidopsis* | 0.071 ± 0.049 |  | 0.157 ± 0.073 | 0.067 ± 0.007 | 0.046 ± 0.022 |  | 0.048 ± 0.018 | 0.013 ± 0.009 | 0.008 ± 0.004 |
|  | Rice | 0.127 ± 0.042 |  | 0.067 ± 0.012 | 0.042 ± 0.004 | 0.059 ± 0.019 |  | 0.070 ± 0.023 | 0.024 ± 0.013 | 0.006 ± 0.004 |
|  |  |  |  |  |  |  |  |  |  |  |

**Table S5.** Levels of PA molecular species in leaves of *Arabidopsis* and rice after various low-temperature treatments. “C” represents “control”, “T” indicates samples taken immediately after each cold treatment, and “R1”and “R3” indicate samples taken after post treatment recovery culture for 1 and 3 days, respectively. Values are means ± S.D. (n=4 or 5).

| PA species | Plant species |  |  |  |  |  |  |  |  |  |
| --- | --- | --- | --- | --- | --- | --- | --- | --- | --- | --- |
|  |  |  |  | *Arabidopsis* (-6 °C)/Rice (10 °C) | | |  | *Arabidopsis* (-12 °C)/Rice (4 °C) | | |
|  |  | C |  | T | R1 | R3 |  | T | R1 | R3 |
|  |  |  |  |  |  |  |  |  |  |  |
|  |  | Lipid content (nmol per mg dry weight) | | | | | | | | |
| **32:0** | *Arabidopsis* | 0.00 ± 0.00 |  | 0.00 ± 0.00 | 0.00 ± 0.01 | 0.00 ± 0.00 |  | 0.04 ± 0.01 | 0.05 ± 0.02 | 0.03 ± 0.01 |
|  | Rice | 0.00 ± 0.00 |  | 0.00 ± 0.00 | 0.00 ± 0.00 | 0.00 ± 0.00 |  | 0.02 ± 0.01 | 0.05 ± 0.01 | 0.04 ± 0.00 |
|  |  |  |  |  |  |  |  |  |  |  |
| **34:1** | *Arabidopsis* | 0.00 ± 0.00 |  | 0.03 ± 0.03 | 0.02 ± 0.02 | 0.01 ± 0.01 |  | 0.31 ± 0.07 | 0.35 ± 0.07 | 0.16 ± 0.06 |
|  | Rice | 0.00 ± 0.00 |  | 0.00 ± 0.01 | 0.00 ± 0.00 | 0.00 ± 0.00 |  | 0.06 ± 0.02 | 0.11 ± 0.03 | 0.07 ± 0.01 |
|  |  |  |  |  |  |  |  |  |  |  |
| **34:2** | *Arabidopsis* | 0.07 ± 0.04 |  | 0.34 ± 0.15 | 0.26 ± 0.12 | 0.11 ± 0.04 |  | 4.81 ± 0.95 | 4.06 ± 1.39 | 1.58 ± 0.81 |
|  | Rice | 0.08 ± 0.03 |  | 0.18 ± 0.05 | 0.05 ± 0.02 | 0.05 ± 0.02 |  | 1.00 ± 0.33 | 1.46 ± 0.34 | 0.86 ± 0.12 |
|  |  |  |  |  |  |  |  |  |  |  |
| **34:3** | *Arabidopsis* | 0.08 ± 0.02 |  | 0.41 ± 0.13 | 0.35 ± 0.15 | 0.16 ± 0.03 |  | 4.52 ± 0.63 | 4.26 ± 0.94 | 1.35 ± 0.63 |
|  | Rice | 0.05 ± 0.02 |  | 0.07 ± 0.02 | 0.01 ± 0.01 | 0.03 ± 0.01 |  | 0.86 ± 0.30 | 1.37 ± 0.27 | 0.61 ± 0.08 |
|  |  |  |  |  |  |  |  |  |  |  |
| **34:4** | *Arabidopsis* | 0.00 ± 0.00 |  | 0.09 ± 0.06 | 0.08 ± 0.04 | 0.01 ± 0.01 |  | 1.85 ± 0.36 | 1.59 ± 0.51 | 0.53 ± 0.35 |
|  | Rice | 0.00 ± 0.00 |  | 0.00 ± 0.00 | 0.00 ± 0.00 | 0.00 ± 0.00 |  | 0.33 ± 0.15 | 0.59 ± 0.12 | 0.24 ± 0.04 |
|  |  |  |  |  |  |  |  |  |  |  |
| **34:5** | *Arabidopsis* | 0.00 ± 0.00 |  | 0.01 ± 0.01 | 0.00 ± 0.01 | 0.00 ± 0.00 |  | 0.11 ± 0.04 | 0.14 ± 0.09 | 0.01 ± 0.01 |
|  | Rice | 0.00 ± 0.00 |  | 0.00 ± 0.00 | 0.00 ± 0.00 | 0.00 ± 0.00 |  | 0.00 ± 0.00 | 0.00 ± 0.00 | 0.00 ± 0.00 |
|  |  |  |  |  |  |  |  |  |  |  |
| **34:6** | *Arabidopsis* | 0.00 ± 0.00 |  | 0.29 ± 0.15 | 0.08 ± 0.08 | 0.00 ± 0.00 |  | 0.95 ± 0.55 | 0.88 ± 0.38 | 0.04 ± 0.03 |
|  | Rice | 0.00 ± 0.00 |  | 0.00 ± 0.00 | 0.00 ± 0.00 | 0.00 ± 0.00 |  | 0.00 ± 0.00 | 0.00 ± 0.00 | 0.00 ± 0.00 |
|  |  |  |  |  |  |  |  |  |  |  |
| **36:2** | *Arabidopsis* | 0.00 ± 0.00 |  | 0.01 ± 0.01 | 0.01 ± 0.01 | 0.00 ± 0.00 |  | 0.21 ± 0.06 | 0.22 ± 0.10 | 0.05 ± 0.05 |
|  | Rice | 0.00 ± 0.00 |  | 0.00 ± 0.00 | 0.00 ± 0.00 | 0.00 ± 0.00 |  | 0.04 ± 0.02 | 0.08 ± 0.02 | 0.05 ± 0.01 |
|  |  |  |  |  |  |  |  |  |  |  |
| **36:3** | *Arabidopsis* | 0.01 ± 0.01 |  | 0.04 ± 0.02 | 0.02 ± 0.01 | 0.01 ± 0.01 |  | 0.63 ± 0.09 | 0.47 ± 0.16 | 0.13 ± 0.07 |
|  | Rice | 0.01 ± 0.01 |  | 0.03 ± 0.02 | 0.00 ± 0.00 | 0.01 ± 0.01 |  | 0.12 ± 0.04 | 0.20 ± 0.07 | 0.08 ± 0.02 |
|  |  |  |  |  |  |  |  |  |  |  |
| **36:4** | *Arabidopsis* | 0.03 ± 0.02 |  | 0.27 ± 0.05 | 0.18 ± 0.07 | 0.05 ± 0.02 |  | 4.02 ± 0.79 | 2.97 ± 1.30 | 0.80 ± 0.54 |
|  | Rice | 0.03 ± 0.01 |  | 0.14 ± 0.03 | 0.03 ± 0.01 | 0.02 ± 0.01 |  | 0.47 ± 0.17 | 0.74 ± 0.17 | 0.36 ± 0.03 |
|  |  |  |  |  |  |  |  |  |  |  |
| **36:5** | *Arabidopsis* | 0.03 ± 0.01 |  | 0.40 ± 0.15 | 0.24 ± 0.12 | 0.10 ± 0.02 |  | 4.87 ± 0.98 | 3.70 ± 0.92 | 0.89 ± 0.57 |
|  | Rice | 0.01 ± 0.01 |  | 0.05 ± 0.01 | 0.01 ± 0.01 | 0.01 ± 0.01 |  | 0.44 ± 0.14 | 0.74 ± 0.19 | 0.30 ± 0.03 |
|  |  |  |  |  |  |  |  |  |  |  |
| **36:6** | *Arabidopsis* | 0.02 ± 0.00 |  | 0.42 ± 0.22 | 0.17 ± 0.12 | 0.05 ± 0.02 |  | 2.43 ± 0.32 | 2.06 ± 0.29 | 0.43 ± 0.18 |
|  | Rice | 0.00 ± 0.00 |  | 0.01 ± 0.01 | 0.00 ± 0.00 | 0.00 ± 0.00 |  | 0.23 ± 0.09 | 0.53 ± 0.12 | 0.19 ± 0.02 |
|  |  |  |  |  | | |  |  | | |
